# Selenium Biofortification Effect on Glucosinolate Content of *Brassica oleracea* var. *italic* and *Eruca vesicaria*

**DOI:** 10.3390/molecules28207203

**Published:** 2023-10-21

**Authors:** Azra Đulović, Katarina Usanović, Lea Kukoč Modun, Ivica Blažević

**Affiliations:** 1 Department of Organic Chemistry, Faculty of Chemistry and Technology, University of Split, Ruđera Boškovića 35, 21000 Split, Croatia; azra@ktf-split.hr (A.Đ.); katarina.usanovic@ktf-split.hr (K.U.); 2 Department of Analytical Chemistry, Faculty of Chemistry and Technology, University of Split, Ruđera Boškovića 35, 21000 Split, Croatia; lea.kukoc-modun@ktf-split.hr

**Keywords:** glucosinolates, broccoli, rocket, 4-(methylseleno)butyl glucosinolate (glucoselenoerucin), UHPLC-MS/MS

## Abstract

Glucosinolates (GSLs) in different plant parts of broccoli (*Brassica oleracea* var. *italic*) and rocket (*Eruca vesicaria*) were analyzed qualitatively and quantitatively before and after treatment with sodium selenate (2 and 5 mM), by their desulfo-counterparts using the UHPLC-DAD-MS/MS technique. Twelve GSLs were detected in broccoli (five aliphatic, one arylaliphatic, and six indolic), where 4-(methylsulfanyl)butyl GSL (glucoerucin) was the main one in the roots (4.88–9.89 µmol/g DW), 4-(methylsulfinyl)butyl GSL (glucoraphanin) in stems (0.44–1.11 µmol/g DW), and 4-hydroxyindol-3-ylmethyl GSL (4-hydroxyglucobrassicin) in leaves (0.51–0.60 µmol/g DW). No GSL containing selenium was detected in the treated broccoli. Ten GSLs were detected in rocket (seven aliphatic and three indolic), where 4-(methylsulfanyl)butyl GSL (glucoerucin) was the main one in the roots (4.50–20.59 µmol/g DW) and 4-methoxyindol-3-ylmethyl GSL (4-methoxyglucobrassicin) in the aerial part (0.57–5.69 µmol/g DW). As a result of induced stress by selenium fertilization, the total GSL content generally increased in both plants. In contrast to broccoli, the roots and the aerial part of the rocket treated with a high concentration of sodium selenate contained 4-(methylseleno)butyl GSL (glucoselenoerucin) (0.36–4.48 µmol/g DW). Although methionine-derived GSLs are the most abundant in both plants, the plants’ ability to tolerate selenate and its regulation by selenoglucosinolate production is species- and growth-stage-dependent.

## 1. Introduction

Selenium (Se) is an important micronutrient required by most living organisms. The difference between deficiency and toxicity is incredibly narrow compared to other micronutrients, with toxic dosages of >400 μg/day and a dietary deficiency of 40 μg/day, which is an emerging global problem [1,2,3]. Plants are important sources of organic Se, as they have the capacity to accumulate inorganic Se or metabolites and store them as organic Se forms. Selenates (SeO_4_^2−^) and selenites (SeO_3_^2−^) are the most significant inorganic forms of Se that are easily taken by plants [4]. Selenate is the most common and fastest-assimilating form of bioavailable Se in alkaline soils with high oxygen content, whereas selenite is more prevalent in anoxic settings with higher acidity and humidity [4]. Plants usually intake selenate, which is reduced, incorporated into organic compounds (selenomethionine, selenocysteine) and further transported through the food chain, and finally decomposed and excreted from the organism, thus completing the biogeochemical cycle of Se [5]. Se levels in soil generally reflect its presence in foods and, therefore, its availability to humans. With an estimated mean Se content in human soft tissues of 110 μg/kg, it was found that the Se intake status in Europe was low, which means that the Se levels in European soils are not sufficient [1,3]. Therefore, biofortification is an important strategy to increase Se in the edible parts of plants.

Based on their ability to accumulate Se, plants can be categorized into three main groups: non-accumulators (accumulate less than 100 mg Se/kg dry weight, DW), secondary accumulators or accumulators (accumulate up to 1000 mg Se/kg DW), and hyperaccumulators (accumulate over 1000 mg Se/kg DW) [6]. The ability to hyperaccumulate Se appears to have evolved within the Asteraceae, Brassicaceae, and Fabaceae families [6]. Broccoli (*Brassica oleracea* var. *italica*) and rocket (*Eruca vesicaria*) are members of Brassicaceae family, which is the largest family in the order Brassicales. Some known Se secondary accumulators include several plants of the *Brassica* genus, such as *Brassica juncea*, *Brassica napus*, and *Brassica oleracea* var. *italic*, whereas Se-hyperaccumulators include plants of the *Cardamine* genus (*Cardamine hupingshanensis*, *Cardamine violofolia*) and *Stanleya* genus (*Stanleya pinnata*, *Stanleya bipinnata*) [6,7]. 

**Figure 1:**
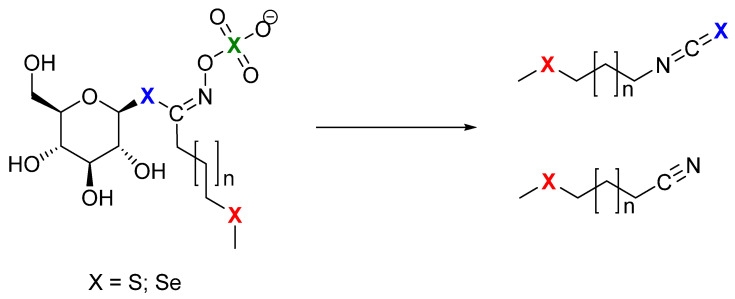
Possible sites (X) for selenium to replace sulfur: green—incorporated in sulfate group; blue—incorporated in thioglucose, and then it is selenoGSL, which produces isoselenocyanides; red—incorporated in side chain, and then it is selenoMet-derived GSL, as breakdown results in isothiocyanates.

Glucosinolates (GSLs) are well-defined specialized plant metabolites containing sulfur and nitrogen which represent the molecular tags of plants from the Brassicaceae family. Generally, they are found in 16 families of the order Brassicales, as well as several plants outside of this order, such as the genus *Drypetes* (family Putranjivaceae, order Malpighiales) and *Rinorea* genus (family Violaceae, order Malpighiales) [8,9]. GSLs can be divided into three classes based on the structure of various amino acid precursors: aliphatic GSLs derived from methionine, isoleucine, leucine, or valine; arylaliphatic GSLs derived from phenylalanine or tyrosine; and indole GSLs derived from tryptophan. Only 90 of the 139 GSLs discovered in the plant kingdom have been thoroughly described by relevant spectroscopy techniques (MS, NMR) to date [10,11,12]. Se-biofortification influences GSL production with possible exchange positions of sulfur (S) by selenium (Se) in three places in the side chain, in thioglucose, and in the sulfate group (Figure 1) [7]. 

Several crop species have been biofortified with Se in field or greenhouse experiments where various Se sources and techniques were used. According to Stewart et al. (1974), sinigrin, which was isolated from horseradish (*Armoracia rusticana*) after receiving Na_2_^75^SeO_4_, was shown to contain Se in its structure [13]. After three weeks at high Se concentrations (100 ppm) in water-cultured specimens of the Se-tolerant species desert princes’ plume (*Stanleya pinnata*), Bertelsen et al. (1988) detected trace quantities of GSL containing Se named as but-3-enylselenoglucosinolate, whereas those grown in environments with lower, more typical Se amounts did not contain this compound [14]. Additionally, no such selenoglucosinolates were found in garden cress (*Lepidium sativum*) and horseradish (*Armoracia rusticana*) [14]. This selenoglucosinolate, originally defined as a compound in which the Se atom is replaced by an S atom in the anomeric position in the GSL structure, is converted into isoselenocyanates by enzymatic hydrolysis [14]. Since the formation of isoselenocyanates rather than isothiocyanates significantly departs from the characteristics of GSLs, they are, by definition, not GSLs (Figure 1).

Matich et al. (2012) treated broccoli (*B. oleracea* var. *italica*), cauliflower (*B. oleracea* L.), and forage rape (*B. napus*) with elevated sodium selenate and examined them for the presence of organoselenides. Based on the analysis of broccoli and cauliflower florets, and the roots of forage rape, the discovery of putative GSL hydrolysis products using synthesized standards confirmed by MS and NMR, as well as by LC-MS analysis, three Se-Met artificially derived GSLs of the general type (methylseleno)alkyl GSL are accepted: 3-(methylseleno)propyl GSL (glucoselenoibervirin **[144]**), 4-(methylseleno)butyl GSL (glucoselenoerucin, **[145]**), and 5-(methylseleno)pentyl GSL (glucoselenoberteroin, **[146]**) [15]. This analysis showed that Se is preferentially incorporated into the methylselenyl moiety rather than the sulfate or *β*-thioglucose groups. A further Se-biofortification study by Matich et al. (2015) using HPLC-MS/MS tentatively indicated glucoselenoraphanin in broccoli (*B. oleracea* var. *italica*) florets with Se envisioned in the side chain and glucoselenonasturtiin in forage rape (*B. napus*) roots, but with Se, that could not be envisioned to be on the side chain, suggesting a selenoGSL sensu Bertelsen et al. [14,16]. 

Black mustard (*Brassica nigra*) seeds grown on naturally Se-rich soils in the Punjab region of India, where the Se soil content ranges from 2 to 7 mg/kg, were studied by Ouerdane et al. (2013) [17]. Using HPLC ESI Orbitrap MS(/MS) and GC APCI TQ MS/MS for the extract analysis, among over 30 Se species, they detected the presence of “selenoglucosinolates”, namely glucoselenoibervirin and glucoselenoerucin, as well as their corresponding degradation products 3-(methylseleno)propylisothiocyanate and 4-(methylseleno)butanenitrile, i.e., 4-(methylseleno)butylisothiocyanate and 5-(methylseleno)pentanenitrile, respectively. To avoid confusion with selenoglucosinolates according to Bertelsen et al. [14], GSLs with Se in the side chain are suggested to be referred to as selenoMet-derived GSLs [8]. The presence of Se in the side chains is fully compatible with the definition of GSLs because they form isothiocyanates and not isoselenocyanates as hydrolysis products (Figure 1). 

The effects of Se-fertilization on GSL production in a radish (*Raphanus sativus*) have been studied by McKenzie et al. (2019) throughout a period of five developmental phases (from seed to fully developed salad greens). With the double bond geometry still unclear, they used tandem mass spectrometry to determine the existence of a novel Se-containing GSL, 4-(methylseleno)but-3-enyl GSL (also named selenoglucoraphenin) [18]. By using GC-MS, two similar isothiocyanates of 4-(methylseleno)but-3-enyl isothiocyanate were tentatively identified as (*E*/*Z*?) isomers. Se-biofertilization of mature radish led to the presence of selenoglucosinolates in the seed [18]. 

The aim of this study is to investigate the influence of Se intake and its metabolism, with special emphasis on the identification and quantification of GSLs and selenoMet-derived GSLs. For this purpose, cultivated broccoli (*Brassica olaracea* var. *italic*) and rocket (*Eruca vesicaria*) were watered with sodium selenate salt solutions (Na_2_SeO_4_) of different concentrations for 35 days and GSLs were analyzed by UHPLC-DAD-MS/MS according to the ISO 9167-1 official method [19]. 

## 2. Results and Discussion

### 2.1. Effect of Selenium Treatment on Broccoli 

*Brassica oleracea* var. *italica*, a young plant which, after 18 days, had no developed flowers or florets, was exposed to Na_2_SeO_4_ concentrations of 2 mM and 5 mM over a period of 35 days. During that time period, the plants did not exhibit any signs of decay of the existing plant parts throughout cultivation. The detrimental influence of selenate solution on the aerial part’s growth was notable when compared to the reference plant’s height, which was 33 cm, and in comparison to 25 cm of the plant exposed to 5 mM selenate solution (Figure S1, Table S1). Se-biofertilization had a detrimental effect on root growth, as evidenced by the fact that the reference plant’s measured roots were 24 cm long compared to 6 cm length of roots watered with the highest Na_2_SeO_4_ concentration (Figure S1, Table S1). The symptom of Se exposure is a reduction in root elongation. Selenate treatment was shown to decrease cytokinin oxidase transcript levels. Cytokinin oxidases in *Arabidopsis* are known to catalyze the irreversible degradation of the hormone cytokinin, in which accumulation in the root tips may partly be responsible for the root meristem shortening [20,21,22]. A previous report by Adamopoulou et al. included the study of Se toxicity on broccoli florets grown hydroponically in a greenhouse for 12 weeks [23,24]. The plants were fortified with two different concentrations of sodium selenate (1.5 mM and 3.0 mM) between the 5^th^ and 10^th^ week. Although broccoli florets grown in the presence and absence of sulfur had the same weight, the absence of sulfur also led to increased Se toxicity, which reduced weight by up to 65%. Also, the leaves’ optical characteristics revealed no visible signs of the biofortification [23,24]. Tian et al. reported the influence of selenium treatment on broccoli growth and showed that when S nutrition was low, Se was particularly harmful to plants and dramatically reduced plant sizes. They proposed that the Se toxicity could be counteracted by increasing sulfate supplementation, which would likely occur via the decreasing non-specific integration of Se into proteins and altering the redox system [25].

**Figure 2:**
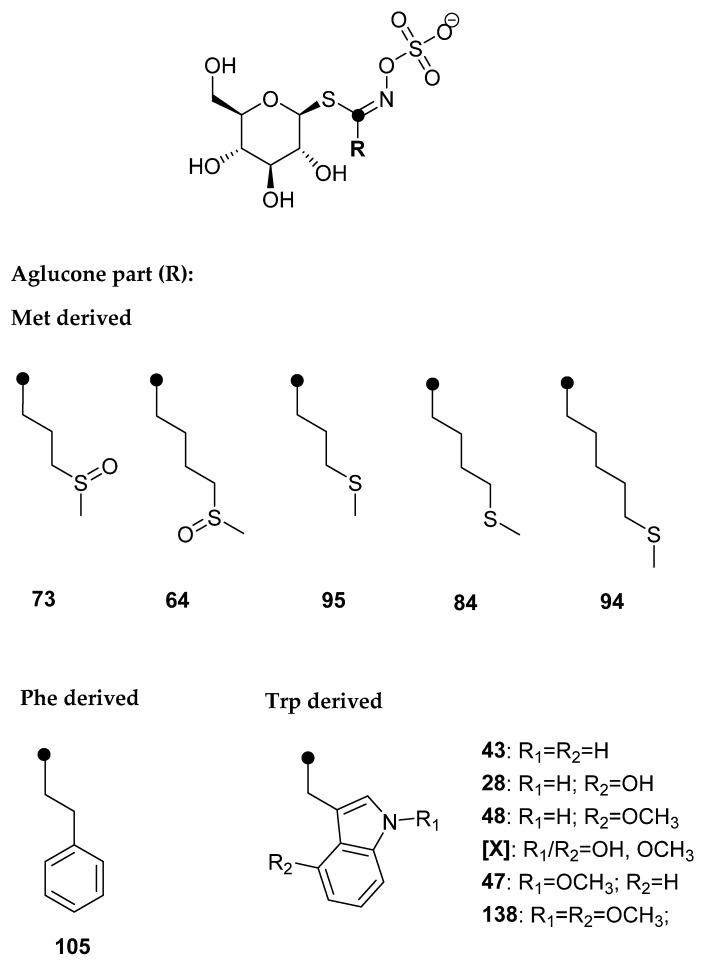
Structures of the GSLs identified in *Brassica oleracea* var. *italica* (*cf*. Table 1).

GSLs were qualitatively and quantitatively analyzed via desulfated forms using UHPLC-DAD-MS/MS, and the results are presented in Table 1 and Figure 2, as well as in the Supplementary Materials (Figures S3–S6).

Five Met-derived desulfoGSLs (dGSLs) with sulfur in the side chain, one Phe-derived dGSL, and six Trp-derived dGSLs were identified. Based on the obtained [M+Na]^+^, no GSLs with Se in the structure were detected. At *t*_R_ = 8.30 min, [M + Na]^+^ = 437 was observed, which was assumed to be a hitherto unidentified dGSL, and was labeled **[X]**. The UV spectrum of dGSL indicated that it is an indole GSL, and the MS^2^ spectrum was recorded in order to identify the characteristic fragments (Figure S6). Fragments *m*/*z* 185 (“a”, Na^+^ adduct of C_6_H_10_O_5_) and *m*/*z* 274 (“c”, loss of C_6_H_10_O_5_) are observed, which suggested that it is a GSL. The fragment *m*/*z* 389 indicated the loss of the methoxy and hydroxyl groups. Based on this information, hydroxymethoxyglucobrassicin was proposed, since it is known that hydroxylation and the subsequent methylation of the indole ring takes place at positions 1 and 4 (R_1_ and R_2_, respectively, Figure 2) [26]. Namely, the key enzymes are cytochrome P450 monooxygenases of the CYP81F subfamily, which carry out hydroxylation reactions at position 4 or 1 of the indole ring. IGMT enzymes, which belong to the family of plant *O*-methyltransferases, convert hydroxy intermediates into methoxylated products, and the enzyme IGMT5 specific for position 1 of the indole ring was found, i.e., IGMT1-4 enzymes specific for position 4 [27]. 

The aliphatic and indole GSL type represent the major GSL found in the analyzed plant parts, with the roots having the highest GSL content. The total content of GSLs in the roots increased for both solution concentrations added (2 mM and 5 mM) when compared to the control and differed significantly (*p* ≤ 0.05). Glucoerucin (**84**), as the main GSL in roots, significantly increased from 4.88 µmol/g DW to 9.05 and 9.89 µmol/g DW, respectively. 

Several studies have reported the existence of Se-GSLs in certain plant parts, such as florets and roots, after the Se-biofertilization of GSL-containing plants. Matich et al. (2012, 2015) showed the presence of Se-GSLs in biofortified broccoli florets [15,16]. However, the same plant in our study did not reach this stage of development (no florets) and no Se-GSLs were detected. According to Tian et al., the total GSL contents of broccoli sprouts determined by glucose from GSL hydrolysis were not significantly changed by the impacts of Se treatments (100 µmol/L selenite and selenate) [25]. However, since the analysis was not conducted in accordance with the ISO 9167 standard method [19], this should only be accepted cautiously.

### 2.2. Effect of Selenium Treatment on Rocket 

*Eruca vesicaria* (rocket), a 24-day old plant, was also exposed to Na_2_SeO_4_ concentrations of 2 mM and 5 mM over a period of 35 days. A similar detrimental influence of Se-biofortification on morphological characteristics was observed for rocket as in broccoli. When sodium selenate (5 mM) was applied, the plant’s height decreased from ca. 23 cm to 8 cm and its root length from ca. 15 cm to 10 cm. In this case, the aerial part biomass decreased, and the beginning of decay was observed for the highest concentration (Figure S2, Table S2).

GSLs were qualitatively and quantitatively analyzed via desulfated forms using UHPLC-DAD-MS/MS, and the results are presented in Table 2 and Figure 3 and Figure 4, as well as in the Supplementary Materials (Figures S7–S12).

**Figure 4:**
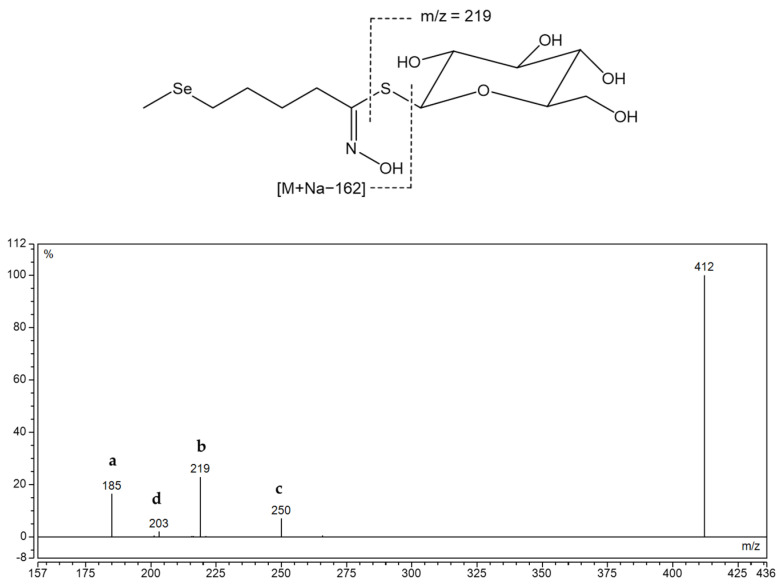
Structure of desulfoglucoselenoerucin **d[145]** and MS^2^ spectra of its sodium adduct. Fragment types observed, alone or in combination, in MS^2^ spectra desulfoglucosinolates in positive mode: **a**—Na^+^ adduct of anhydroglucose, C_6_H_10_O_5_ (at *m*/*z* 185) or an acyl derivative; **b**—Na^+^ adduct of thioglucose, C_6_H_11_O_5_SH (at *m*/*z* 219) or an acyl derivative; **c**—Loss of anhydroglucose (*m*/*z* 162) or an acyl derivative; **d**—Na^+^ adduct of glucose, C_6_H_12_O_6_ (at *m*/*z* 203) [28].

Six methionine-derived GSLs and three indole ones were identified in the rocket. By comparing the chromatograms obtained for the root and aerial component, it was discovered that Se-treated samples exhibit a signal that is not present in the reference plant, particularly in the root. Its [M+Na]^+^ was 412 at *t*_R_ = 7.6 min, which did not correspond to previously known GSL of that mass. It was assumed that it is selenomethionine-derived selenoglucosinolate, namely glucoselenoerucin (**[145]**), whose desulfo form structure is given in Figure 4, since the obtained mass corresponds to this dGSL when sulfur is replaced by selenium.

In the MS^2^ spectrum, fragments marked “a”, “b”, “c”, and “d” are present, indicating that Se was not found in the thioglucose part, but in the side chain derived from selenomethionine (Se-Met). Right next to 412, 410 appears, which is assumed to be the isotope of selenium 78, so MS^2^ was obtained for this [M + Na]^+^ and fragment *m*/*z* 185 (a) was also observed (Figure S12). According to previous literature, this selenoglucosinolate was identified for the first time through its desulfated form and its MS^2^ was shown for the first time.

The total GSL content is related mostly to the content of aliphatic GSLs, with the highest found in the roots. The occurrence of this GSL derived from Se-Met can be related to the high content of its sulfur analogue glucoerucin (**84**), and the highest content was obtained in the roots of the plant treated with 2 mM solution, 4.48 µmol/g DW. With the addition of 5 mM sodium solution, rocket showed the signs of decay and the total GSL content decreased, having 14.21 and 2.47 µmol/g DW in the roots and aerial parts, in comparison to the control having 32.34 and 13.24 µmol/g DW, respectively, and differed significantly (*p* ≤ 0.05). Consequently, the content of GSL **[145]** decreased. Dall’Acqua et al. studied the Se-biofortification of two rocket species (*Eruca sativa* = *E. vesicaria* and *Diplotaxis tenuifolia*) grown at 0–40 µM Na_2_SeO_4_ in hydroponics and also showed a GSL decrease [29]. Contrary to our investigation, selenoglucosinolates have not been reported in these species. Also, they showed that selenium treatment reduced cysteine and methionine content, and glutathione (GSH), whose precursor is cysteine, also showed a trend toward reduction; thus, a decrease in GSLs, also observed in this study, may be explained by the negative effects of selenate on aminoacids biosynthesis, during which selenoamino acids can be formed and be incorporated into proteins and/or form selenoGSLs [30].

## 3. Materials and Methods

### 3.1. Materials and Reagents

4-(Methylsulfinyl)butyl GSL (glucoraphanin, **64**), 4-(methylsulfanyl)butyl GSL (glucoerucin, **84**), 5-(methylsulfanyl)pentyl GSL (glucoberteroin, **94**), 9-(methylsulfinyl)nonyl GSL (glucoarabin, **68**), 10-(methylsulfinyl)decyl GSL (glucocamelinin, **65**), 2-phenylethyl GSL (gluconasturtiin, **105**), indol-3-ylmethyl GSL (glucobrassicin, **43**), 4-hydroxyindol-3-ylmethyl GSL (4-hydroxyglucobrassicin, **28**), 4-methoxyindol-3-ylmethyl GSL (4-methoxyglucobrassicin, **48**), and *N*-methoxyindol-3-ylmethyl GSL (neoglucobrassicin, **47**) were purchased from Phytoplan Diehm & Neuberger GmbH (Heidelberg, Germany). 4-Hydroxybenzyl GSL (glucosinalbin, **23**) was isolated from *Sinapis alba* seeds and 3-(methylsulfinyl)propyl GSL (glucoiberin, **73**) was isolated from *Anastatica hierochuntica*, both confirmed by NMR. All other chemicals and reagents were of analytical grade.

### 3.2. Plant Growth and Harvesting

Young plants of *Brassica oleracea* var. *italica* (broccoli), one month old and with a total height of 18 cm (root 4 cm), were transplanted into pots (one plant per pot) (dimensions, (w × d × h) 12 × 12 × 10 cm) with soil Potgrond H (Potgrond H 70, Klasmann-Deilmann GmbH, Geeste, Germany) (pH = 6.0) and watered with tap water during the next fifteen days in order to adapt. On the fifteenth day after transplantation, the height of the above-ground part was measured, which was 18 cm, and the height of the stem was 10 cm. The reference plant was watered only with water, whereas the remaining two plants were watered with prepared solutions of sodium selenate with a concentration of 2 mM and 5 mM (three repetition per experiment). During cultivation, the plants were watered with tap water for 35 days, and they were biofortified twice a week with a 20 mL selenate solution. We estimate this selenium application to be equivalent to ca. 22 and 55 kg/ha, respectively, which is considerably higher than the 3–76 g/ha applied to agricultural and forage crops [15,16]. The growth and development of the above-ground part were recorded throughout the period, and on the 35^th^ day from the beginning of watering, the length of the roots and the height of the above-ground part were measured.

*Eruca vesicaria* (rocket) was grown from the seeds of the Italian distributor Impex s.r.l. cement, obtained from the Sjeme store, Split, Croatia. Seeds (100 mg) were placed in each cultivation pot (n = 3) with soil Potgrond H and germination began on the 3rd day after watering. On the 10th day, the plants were transplanted into three larger pots (dimensions, (w × d × h) 12 × 12 × 10 cm) and on the 24^th^ day after sowing, watering with sodium selenate solutions was started. The height of the aerial part of the plants before watering was 7.5 cm, and the length of the roots was 3 cm.

After collection, all plants were washed with tap water and lyophilized. They were divided into the root and aerial part for the purpose of the qualitative and quantitative analysis of GSLs. The lengths of plant parts were measured by a ruler with an accuracy of 0.1 cm, for the control and each treatment (*n* = 3).

### 3.3. Isolation and Chemical Analysis

#### 3.3.1. Isolation of Desulfoglucosinolates

GSLs were extracted as previously reported [31]. Freeze-dried plant parts were ground to a fine powder, from which 100 mg was extracted for 5 min at 80 °C in 2 × 1 mL MeOH/H_2_O (70:30 *v*/*v*) to inactivate the endogenous myrosinase. Each extract (1 mL) was loaded onto a mini-column filled with 0.5 mL of DEAE-Sephadex A-25 anion-exchange resin (GE Healthcare) conditioned with 25 mM acetate buffer (pH 5.6). After washing the column with 70% MeOH and 1 mL of ultrapure water, the optimal conditions for desulfation were set by adding a buffer solution. Each mini-column was loaded with 20 μL (0.35 U/mL) of purified sulfatase and left to stand for 18 h at room temperature. The desulfoGSLs were then eluted with 1.5 mL of ultra-pure H_2_O, lyophilized, and diluted to 1 mL. The samples were stored at −20 °C until further analysis by UHPLC-DAD-MS/MS.

#### 3.3.2. UHPLC-MS/MS Analysis

Analysis was performed on UHPLC-MS/MS (Ultimate 3000RS with TSQ Quantis MS/MS detector, Thermo Fisher Scientific, Waltham, MA, USA) using a Hypersil GOLD column (3.0 µm, 3.0 × 100 mm, Thermo Fisher Scientific, Waltham, MA, USA). A gradient consisting of solvent A (50 μM NaCl in H_2_O) and solvent B (acetonitrile:H_2_O 30:70 *v*/*v*) was applied at a flow rate of 0.5 mL/min as follows: 0.14 min 96% A; 7.84 min 14% A; 8.96 min 14% A; 9.52 min 5% A; 13.16 min 5% A; 13.44 min 96% A; 15.68 min 96% A. The column temperature was held at 15 °C for *E. vesicaria* dGSLs and 25 °C for *B. oleracea* var. *italica* dGSLs and the injection volume was 5 µL. The electrospray interface was an H-ESI source operating with a capillary voltage of 3.5 kV at 350 °C. The system was operated in the positive ion electrospray mode.

Qualitative analysis was performed by the manual examination of chromatograms and MS^2^ spectra of each peak of desulfoglucosinolate as sodium adduct ([M+Na]^+^). The amount of GSLs was quantified using a calibration curve of pure desulfosinigrin solution (range from 0.14 to 1.4 mM) and RPFs for each individual desulfoGSL [31]. The RPF values for quantification of desulfoGSLs were as follows: RPF 1.07 for 3-(methylsulfinyl)propyl GSL (glucoiberin, **73**) and 4-(methylsulfinyl)butyl GSL (glucoraphanin, **64**); 0.80 for 3-(methylsulfanyl)propyl GSL (glucoibervirin, **95**); 1.04 for 4-(methylsulfanyl)butyl GSL (glucoerucin, **84**); 0.50 for glucosinalbin (**23**); 0.95 for gluconasturtiin (**105**); 0.29 for glucobrassicin (**43**); 0.28 for 4-hydroxyglucobrassicin (**28**); 0.25 for 4-methoxyglucobrassicin (**48**); 0.20 for N-methoxyglucobrassicin (**47**) and 1,4-dimethoxyindol-3-ylmethyl GSL (**138**) [32,33]; arbitrary RPF 0.25 for hydroxymethoxyglucobrassicin (**[X])**, and 1.0 for 5-(methylsulfanyl)pentyl GSL (glucoberteroin, **94**), 9-(methylsulfinyl)nonyl GSL (glucoarabin, **68**), 9-(methylsulfonyl)nonyl GSL (**79**), 10-(methylsulfinyl)decyl GSL (**65**), 4-(methylseleno)butyl GSL **[145]**, 4-(β-d-glucopyranosyldisulfanyl)butyl GSL **(135)**, and dimeric 4-mercaptobutyl GSL (**134**). The bold numbers are related to the GSL number given in the review paper by Blažević et al. [10].

### 3.4. Statistical Analysis

One-way analysis of variance (ANOVA) was performed with SPSS software, version 25.0 (IBM Corporation, New York, NY, USA), in order to determine the difference between treatments. First, the data were tested for normal distribution and log-transformed for further analysis. One-way ANOVA was performed if the requirement of homogeneity of variance was fulfilled; otherwise, Welch correction was performed first. If there was a statistically significant effect between different treatments, Tukey’s honest significant test was performed with a significance level of *p* ≤ 0.05.

## 4. Conclusions

The morphological changes and changes in GSL content in broccoli and rocket were used to track the stress caused by selenate. Morphological alterations were detected in the form of decreased root development, which can be explained by the competing action of sulfate and selenate. Both plants’ principal GSLs were produced from methionine biosynthesis, with the major GSL being 4-(methylsulfanyl)butyl GSL (glucoerucin, **84**). However, only rocket biosynthesized the Se analogue of **84**, 4-(methylseleno)butyl GSL (glucoselenoerucin, **[145]**). Se was determined in the side chain based on distinctive fragmentations in the MS^2^ spectrum, which was recorded and analyzed for the first time for this desulfoselenoglucosinolate. Although other Met-derived GSLs with sulfur in the side chain have been identified, no Se analogues have been found. This study emphasizes the importance of plant tolerance to selenate and the growth stage, such as in broccoli, where biofortification should begin as soon as the florets begin to emerge from the meristerm and not before.

## Figures and Tables

**Figure 3:**
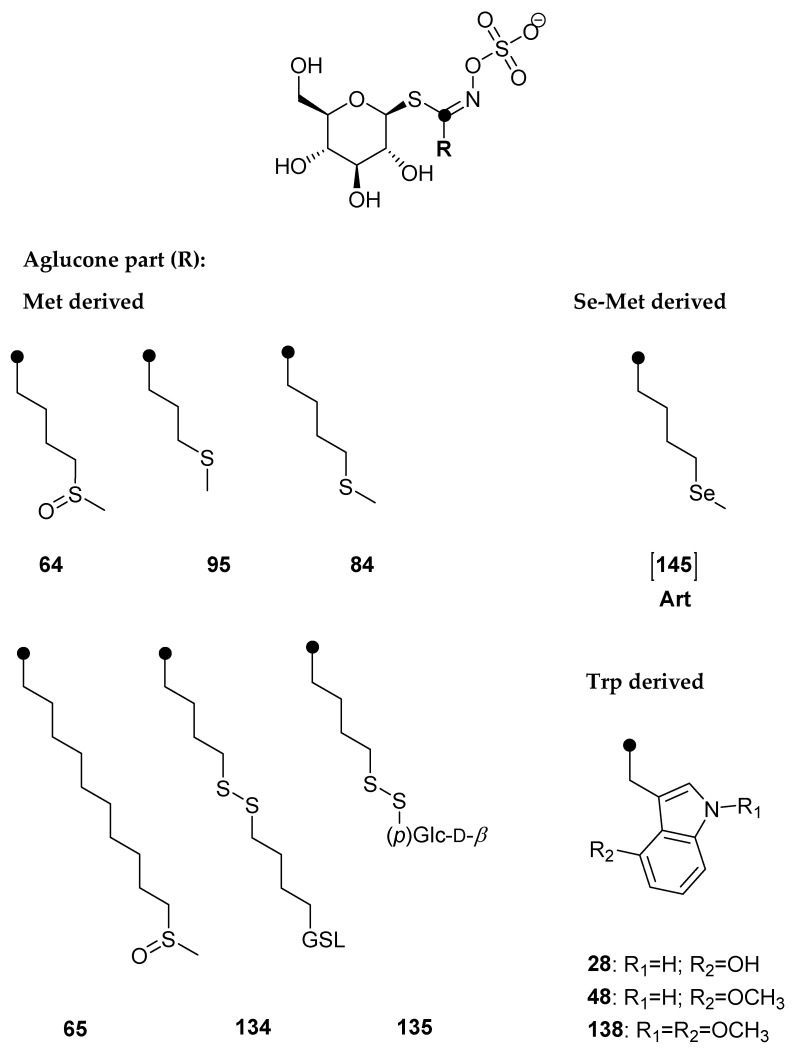
Structures of the GSLs identified in *Eruca vesicaria* (*cf*. Table 2).

**Table 1:** Glucosinolate content (µmol/g DW) in *Brassica oleracea* var. *italica*.

No.	Glucosinolate	Formula (desulfo)	Desulfo M	Desulfo [M + Na]^+^	Reference			Na_2_SeO_4_ Solution					
								2 mM			5 mM		
					Root	Stem	Leaf	Root	Stem	Leaf	Root	Stem	Leaf
	Met derived												
**73**	3-(Methylsulfinyl)propyl GSL (glucoiberin) ^a^	C_11_H_21_NO_7_S_2_	343	366	tr	tr	n.d.	tr	tr	n.d.	tr	tr	n.d.
**64**	4-(Methylsulfinyl)butyl GSL (glucoraphanin) ^a^	C_12_H_23_NO_7_S_2_	357	380	2.09 ± 0.12 ^A^	1.11 ± 0.06 ^D^	tr	1.40 ± 0.13 ^E^	0.58 ± 0.04 ^B^	tr	3.68 ± 0.87 ^C^	0.44 ± 0.12 ^E^	tr
**95**	3-(Methylsulfanyl)propyl GSL (glucoibervirin) ^b^	C_11_H_21_NO_6_S_2_	327	350	n.d.	n.d.	n.d.	n.d.	n.d.	n.d.	1.03 ± 0.12	n.d.	n.d.
**84**	4-(Methylsulfanyl)butyl GSL (glucoerucin) ^a^	C_12_H_23_NO_6_S_2_	341	364	4.88 ± 0.72 ^A^	0.13 ± 0.00 ^D^	n.d.	9.05 ± 0.12 ^B^	0.11 ± 0.00 ^E^	n.d.	9.89 ± 0.57 ^B^	tr	n.d.
**94**	5-(Methylsulfanyl)pentyl GSL (glucoberteroin) ^a^	C_15_H_21_NO_6_S	355	378	tr	n.d.	tr	n.d.	n.d.	n.d.	tr	n.d.	n.d.
	Phe derived												
**105**	2-Phenylethyl GSL (gluconasturtiin) ^a^	C_11_H_21_NO_7_S_2_	343	366	tr	n.d.	tr	tr	n.d.	n.d.	0.31 ± 0.00	n.d.	n.d.
	Trp derived												
**43**	Indol-3-ylmethyl GSL (glucobrassicin) ^a^	C_16_H_20_N_2_O_6_S	368	391	0.68 ± 0.14 ^A^	0.07 ± 0.00 ^D^	0.31 ± 0.04 ^G^	2.34 ± 0.13 ^B^	0.55 ± 0.12 ^E^	0.09 ± 0.00 ^H^	1.20 ± 0.18 ^C^	0.25 ± 0.00 ^F^	0.58 ± 0.12 ^I^
**28**	4-Hydroxyindol-3-ylmethyl GSL (4-hydroxyglucobrassicin) ^a^	C_16_H_20_N_2_O_7_S	384	407	0.62 ± 0.10 ^A^	tr	0.60 ± 0.20 ^G^	0.78 ± 0.19 ^A^	0.22 ± 0.09	0.51 ± 0.15 ^G^	tr	tr	0.57 ± 0.00 ^G^
**48**	4-Methoxyindol-3-ylmethyl GSL (4-methoxyglucobrassicin) ^a^	C_17_H_22_N_2_O_7_S	398	421	3.23 ± 0.43 ^A^	0.15 ± 0.00 ^D^	0.09 ± 0.00	4.23 ± 0.12 ^B^	0.15 ± 0.00 ^D^	0.10 ± 0.00	3.80 ± 0.43 ^A,B^	0.53 ± 0.12 ^E^	0.45 ± 0.00
**[X]**	Hydroxymethoxy- glucobrassicin	C_17_H_22_N_2_O_8_S	414	437	tr	tr	0.24 ± 0.08 ^G^	0.04 ± 0.00	0.04 ± 0.00	0.02 ± 0.00 ^H^	n.d.	n.d.	0.03 ± 0.00 ^H^
**47**	*N*-Methoxyindol-3-ylmethyl GSL (neoglucobrassicin) ^a^	C_17_H_22_N_2_O_7_S	398	421	1.97 ± 0.31 ^A^	0.20 ± 0.05 ^D^	0.04 ± 0.00 ^G^	2.73 ± 0.33 ^B^	0.56 ± 0.12 ^E^	tr	2.43 ± 0.12 ^A,B^	0.20 ± 0.00 ^D^	0.18 ± 0.00 ^G^
**138**	1,4-Dimethoxyindol-3-ylmethyl GSL (1,4-dimethoxyglucobrassicin) ^b^	C_18_H_24_N_2_O_8_S	428	451	n.d.	n.d.	n.d.	n.d.	n.d.	n.d.	tr	n.d.	n.d.
	Total (µmol/g DW)				13.48 ± 1.82 ^J^	1.66 ± 0.11 ^M,N^	1.28 ± 0.32 ^P,R^	20.57 ± 1.02 ^K^	2.21 ± 0.37 ^M,N^	0.72 ± 0.15 ^P,R^	22.34 ± 2.29 ^K^	1.42 ± 0.24 ^M^	1.81 ± 0.12 ^P^

No.—numbers are related to the glucosinolate number given in review paper by Blažević et al. [10]; Desulfo [M + Na]^+^, sodium adduct of desulfoglucosinolate used for GSL identification. ^a^ Compound identified by MS^2^ spectra and *t*_R_ comparison with standard. ^b^ Compound identified by MS^2^ spectra and t_R_ comparison with the literature. The structures are shown in Figure 2. All chromatograms are given in Figures S3–S5, and MS^2^ spectra of all identified desulfoGSLs are given in Figure S10, except of hypothesized dGSL designated as **[X]**, the MS^2^ spectra of which are given in Figure S6. GSL—glucosinolate; tr—traces (<0.01 µmol/g DW); n.d.—not detected; DW—dry weight of plant material. Data are expressed as the mean value ± standard error (*n* = 3). Statistical analyses are performed by one-way ANOVA followed by Tukey’s honest significant test. Mean values with a different letter differ significantly (*p* ≤ 0.05). Statistical significance: root—letters ^A–C^, stem—letters ^D–F^, leaf—letters ^G–I^, root total—letters ^J,K^ stem total—letters ^M,N^, leaf total ^P,R^.

**Table 2:** Glucosinolate content (µmol/g DW) in cultivated *Eruca vesicaria*.

No.	Glucosinolate (GSL)	Formula (desulfo)	Desulfo M	Desulfo [M + Na]^+^	Reference		Na_2_SeO_4_ solution			
							2 mM		5 mM	
					Root	Aerial Part	Root	Aerial Part	Root	Aerial Part
	Met derived									
**64**	4-(Methylsulfinyl)butyl GSL (glucoraphanin) ^a^	C_12_H_23_NO_7_S_2_	357	380	3.23 ± 0.63 ^A^	0.87 ± 0.22 ^D^	1.69 ± 0.34 ^B^	0.95 ± 0.11 ^D^	0.75 ± 0.15 ^C^	0.74 ± 0.12 ^D^
**135**	4-(β-d-Glucopyranosyldisulfanyl)butyl GSL (diglucothiobeinin) ^b^	C_17_H_31_NO_11_S_3_	521	544	tr	1.14 ± 0.24	n.d.	n.d.	n.d.	n.d.
**95**	3-(Methylsulfanyl)propyl GSL (glucoibervirin) ^b^	C_11_H_21_NO_6_S_2_	327	350	1.88 ± 0.33	1.88 ± 0.17	n.d.	n.d.	n.d.	n.d.
**84**	4-(Methylsulfanyl)butyl GSL (glucoerucin) ^a^	C_12_H_23_NO_6_S_2_	341	364	20.59 ± 1.32 ^A^	2.78 ± 0.33 ^D^	17.27 ± 1.12 ^A^	1.30 ± 0.08 ^E^	4.50 ± 0.55 ^B^	0.21 ± 0.05 ^F^
**134**	Dimeric 4-mercaptobutyl GSL ^b^	C_22_H_40_N_2_O_12_S_4_	652	675	4.33 ± 0.62 ^A^	0.20 ± 0.00 ^D^	17.14 ± 0.93 ^B^	1.49 ± 0.22 ^E^	6.78 ± 0.77 ^C^	0.55 ± 0.12 ^F^
**65**	10-(Methylsulfinyl)decyl GSL (glucocamelinin) ^a^	C_18_H_35_NO_7_S_2_	441	464	tr	0.21 ± 0.12	tr	tr	n.d.	tr
	Se-Met derived									
**[145]**	4-(Methylseleno)butyl GSL (glucoselenoerucin)	C_12_H_23_NO_6_SSe	389	412	n.d.	n.d.	4.48 ± 0.55 ^A^	1.24 ± 0.33 ^D^	0.78 ± 0.29 ^B^	0.36 ± 0.10 ^E^
	Trp derived									
**28**	4-Hydroxyindol-3-ylmethyl GSL (4-Hydroxyglucobrassicin) ^a^	C_16_H_20_N_2_O_7_S	384	407	0.23 ± 0.00	n.d.	tr	n.d.	n.d.	n.d.
**48**	4-Methoxyindol-3-ylmethyl GSL (4-Methoxyglucobrassicin) ^a^	C_17_H_22_N_2_O_7_S	398	421	1.16 ± 0.22 ^A^	5.96 ± 1.03 ^D^	0.80 ± 0.11 ^B^	1.64 ± 0.36 ^E^	0.07 ± 0.00 ^C^	0.57 ± 0.12 ^F^
**138**	1,4-Dimethoxyindol-3-ylmethyl GSL (1,4-dimethoxyglucobrassicin) ^b^	C_18_H_24_N_2_O_8_S	428	451	0.92 ± 0.12 ^A^	0.20 ± 0.00	2.06 ± 0.18 ^B^	0.15 ± 0.00	1.33 ± 0.31 ^A^	0.04 ±0.00
	Total (µmol/g DW)				32.34 ± 3.24 ^G^	13.24 ± 2.11 ^J^	43.44 ± 3.23 ^H^	6.77 ± 1.10 ^K^	14.21 ± 2.07 ^I^	2.47 ± 0.51 ^L^

No.—numbers are related to the glucosinolate number given in review paper by Blažević et al. [10]; Desulfo [M + Na]^+^, sodium adduct of desulfoglucosinolate used for GSL identification. ^a^ Compound identified by MS^2^ spectra and *t*_R_ comparison with standard. ^b^ Compound identified by MS^2^ spectra and t_R_ comparison with the literature. The structures are shown in Figure 3. All chromatograms are given in Figures S7 and S8, whereas MS^2^ spectra of all identified sodium adduct of desulfoGSLs are given in Figure S10, except for 4-(methylseleno)butyl desulfoglucosinolate, designated as d**[145]**, for which the UV, MS, and MS^2^ of are given in Figures S11 and S12. GSL—glucosinolate; tr—traces < 0.01 µmol/g DW; n.d.—not detected; DW—dry weight of plant material. Data are expressed as the mean value ± standard error (*n* = 3). Statistical analyses are performed by one-way ANOVA followed by Tukey’s honest significant test. Mean values with a different letter differ significantly (p ≤ 0.05). Statistical significance: root—letters ^A–C^, aerial part—letters ^D–F^, root total—letters ^G–I^, aerial part total—letters ^J–L^.

## Data Availability

Not applicable.

## Supplementary Materials

The following are available online at https://www.mdpi.com/article/10.3390/molecules28207203/s1, Figure S1. Broccoli (*Brassica oleracea* var. *italic*) watered with water (reference) and different concentrations of sodium selenate solution; Table S1. The effect of selenate treatments on *B. oleracea* var. *italic*; Figure S2. Rocket (*Eruca vesicaria*) watered with water (reference) and different concentrations of sodium selenate solution.; Table S2. The effect of selenate treatments on *E. vesicaria*; Figure S3. Chromatograms of desulfoglucosinolates (column temperature 25 °C) obtained from the roots of *Brassicca oleracea* var. *italica* before and after the treatment with Na_2_SeO_4_; Figure S4. Chromatograms of desulfoglucosinolates (column temperature 25 °C) obtained from the stem of *Brassicca oleracea* var. *italica* before and after the treatment with Na_2_SeO_4_; Figure S5. Chromatograms of desulfoglucosinolates (column temperature 25 °C) obtained from the leaf of *Brassicca oleracea* var. *italica* before and after the treatment with Na_2_SeO_4_; Figure S6. MS^2^ spectra at 15V and 20V of desulfo-4-hydroxy-*N*-methoxyglucobrassicin; Figure S7. Chromatograms of desulfoglucosinolates (column temperature 15 °C) obtained from the roots of *Eruca vesicaria* before and after the treatment with Na_2_SeO_4_; Figure S8. Chromatogram of desulfoglucosinolates (column temperature 15 °C) obtained from the aerial part of *Eruca vesicaria* before and after the treatment with Na_2_SeO_4_; Figure S9. Chromatograms of desulfoglucosinolate (dGSL) standards.; Figure S10. MS^2^ spectra of desulfoglucosinolates.; Figure S11. MS and UV spectra of detected desulfoglucoselenoerucin. Figure S12. MS^2^ spectra at 15V of desulfoglucoselenoerucin formed during biofortification (two isotopes of Se—*m*/*z* 410 and 412).

## Sample Availability

Samples of the compounds are available from the authors.
