# Supplementary material for: Selenium Biofortification Effect on Glucosinolate Content of Brassica oleracea var. italic and Eruca vesicaria
Source: Molecules. 2023 Oct 21;28(20):7203. doi: 10.3390/molecules28207203 (PMC10609431; doi:10.3390/molecules28207203)
Supplement: Supplementary file 1 [file molecules-28-07203-s001.zip › molecules-2609761-supplementary.pdf]

molecules

Supplementary Materials

Selenium Biofortification Effect on Glucosinolate Content of *Brassica oleracea* var. *italic* and *Eruca vesicaria*

Azra Đulović [1], Katarina Usanović [1], Lea Kukoč Modun [2], Ivica Blažević [1,*]

[1] Department of Organic Chemistry, Faculty of Chemistry and Technology, University of Split, Ruđera Boškovića 35, 21000 Split, Croatia; azra@ktf-split.hr (A.Đ.); katarina.usanovic@ktf-split.hr (K.U.)
[2] Department of Analytical Chemistry, Faculty of Chemistry and Technology, University of Split, Ruđera Boškovića 35, 21000 Split, Croatia; lea.kukoc-modun@ktf-split.hr
* Correspondence: blazevic@ktf-split.hr

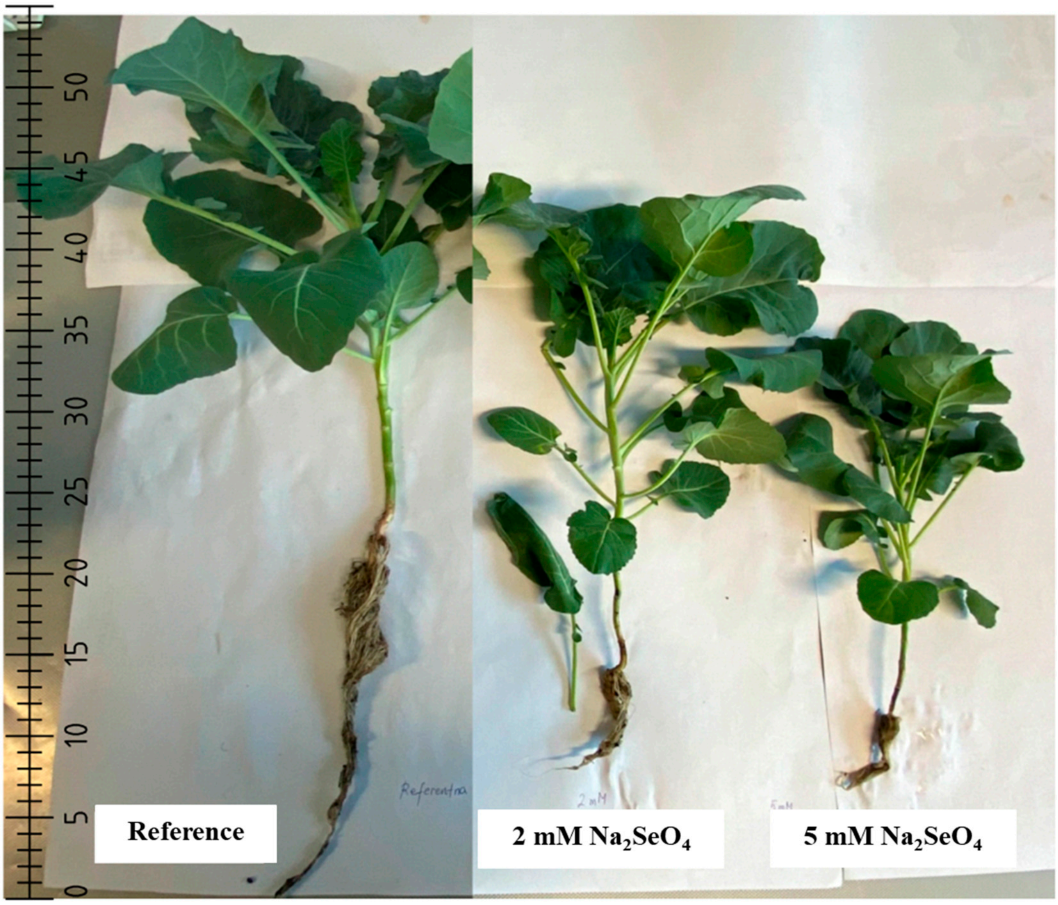

Figure S1. Broccoli (*Brassica oleracea* var. *italic*) watered with water (reference) and different concentrations of sodium selenate solution

Table S1. The effect of selenate treatments on *B. oleracea* var. *italic*

	Control	2 mM SeO_4^{2-}	5 mM SeO_4^{2-}
Root length (cm)	24.0 ± 0.7	7.0 ± 0.5	6.0 ± 0.4
Aerial part length (cm)	33.0 ± 0.6	29.0 ± 0.4	25.0 ± 0.6
Aerial part biomass (g dried weight)	1.69 ± 0.24	1.30 ± 0.15	1.17 ± 0.13

Data are mean ± SD (*n* = 3)

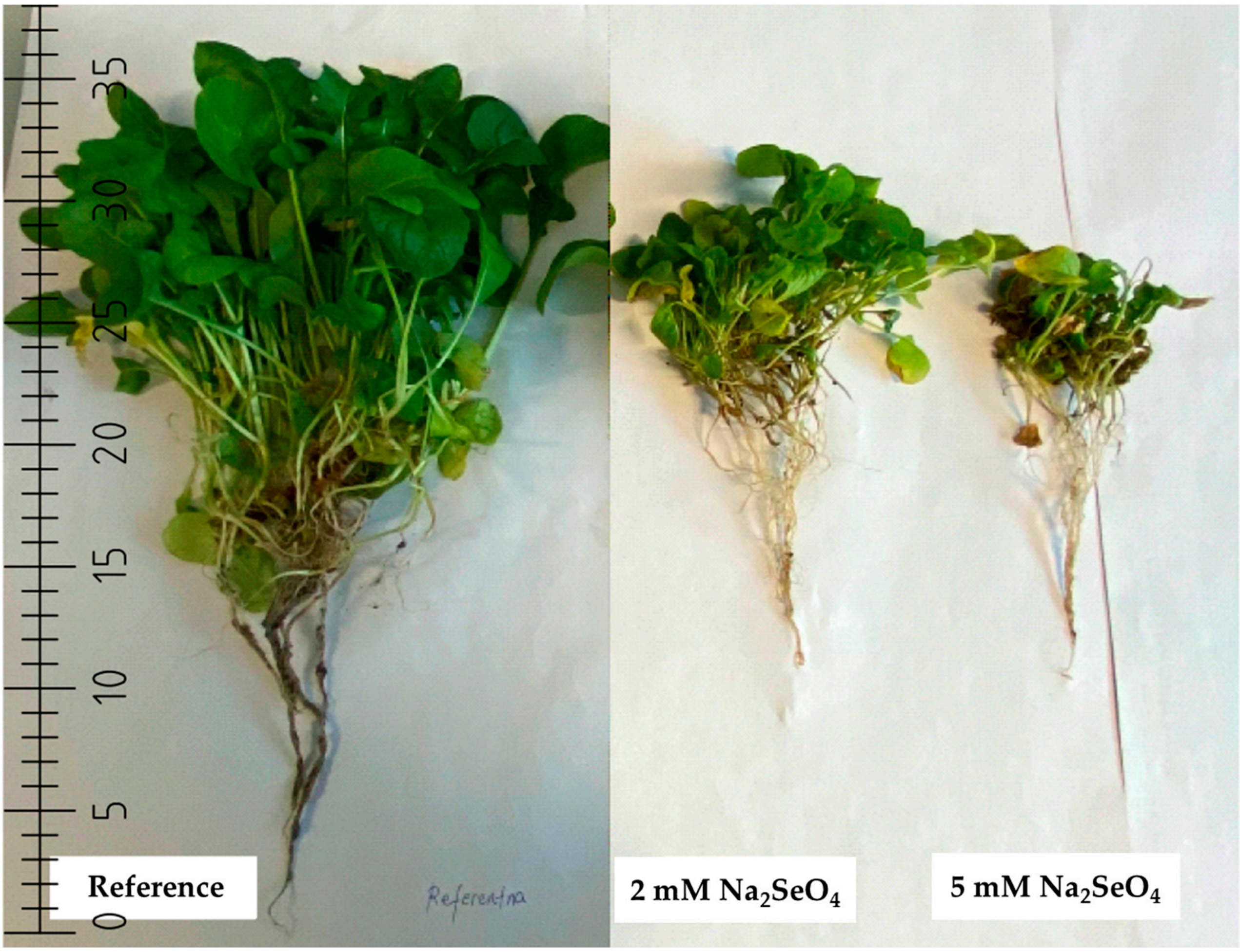

Figure S2. Rocket (*Eruca vesicaria*) watered with water (reference) and different concentrations of sodium selenate solution.

Table S2. The effect of selenate treatments on *E. vesicaria*

	Control	2 mM SeO_4^{2-}	5 mM SeO_4^{2-}
Root length (cm)	15.1 ± 0.4	10.6 ± 0.5	9.7 ± 0.4
Aerial part length (cm)	22.8 ± 0.6	10.2 ± 0.4	7.8 ± 0.6
Aerial part biomass (g dried weight)	1.45 ± 0.24	0.45 ± 0.15	0.42 ± 0.13

Data are mean ± SD (*n* = 3)

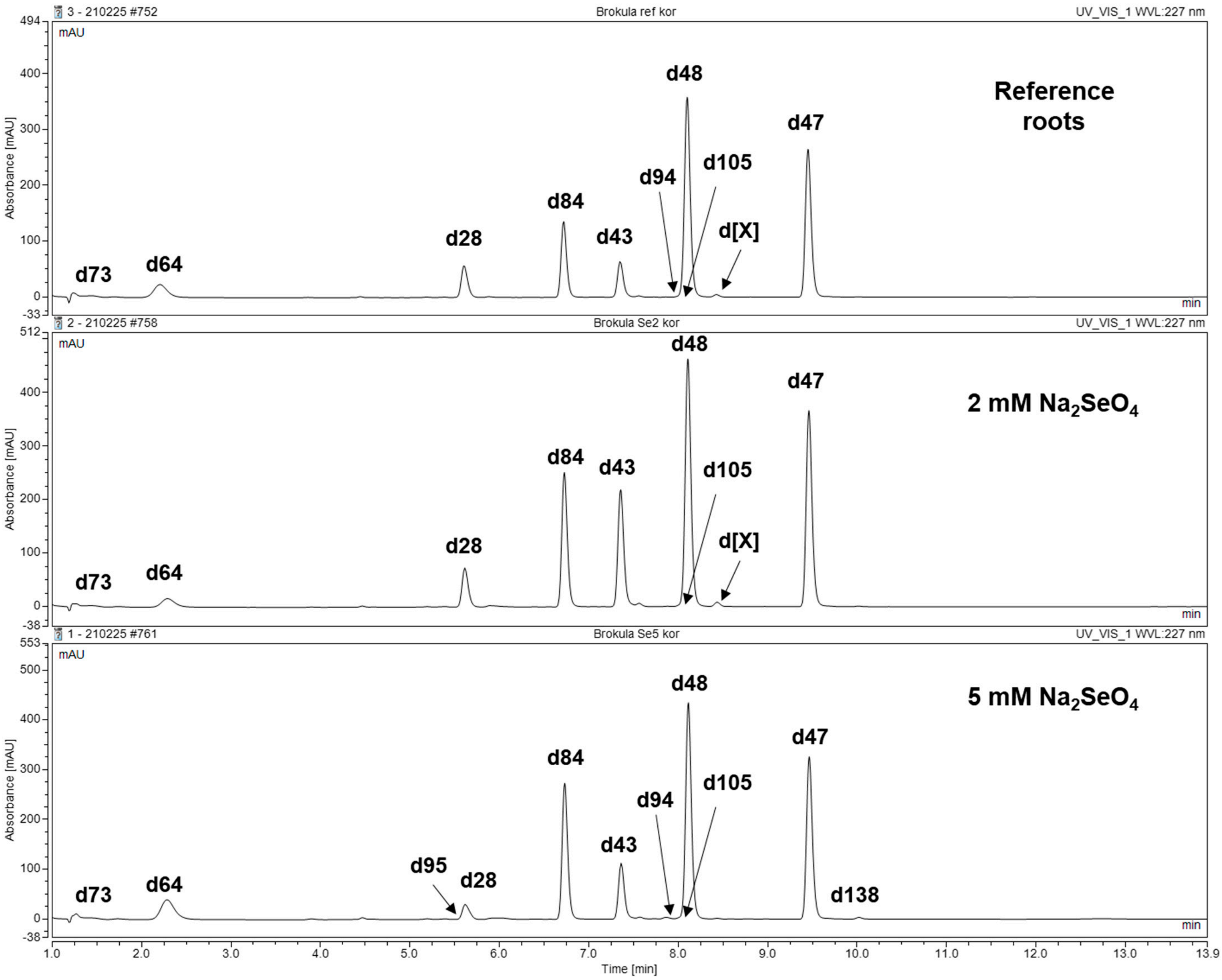

Figure S3. Chromatograms of desulfoglucosinolates (column temperature 25 °C) obtained from the roots of *Brassicca oleracea* var. *italica* before and after the treatment with Na_2SeO_4: **d73** – desulfoglucoiberin; **d64** – desulfoglucoraphanin; **d95** – desulfoglucoibervirin; **d28** –desulfo-4-hydroxyglucobrassicin; **d84** – desulfoglucoerucin; **d43** – desulfoglucobrassicin; **d94** – glucoberteroin**;** **d48** – desulfo-4-methoxyglucobrassicin; **d105** – desulfogluconasturtiin; **d[X]** – hydroxymethoxydesulfoglucobrassicin; **d47** – desulfogluconeobrassicin.

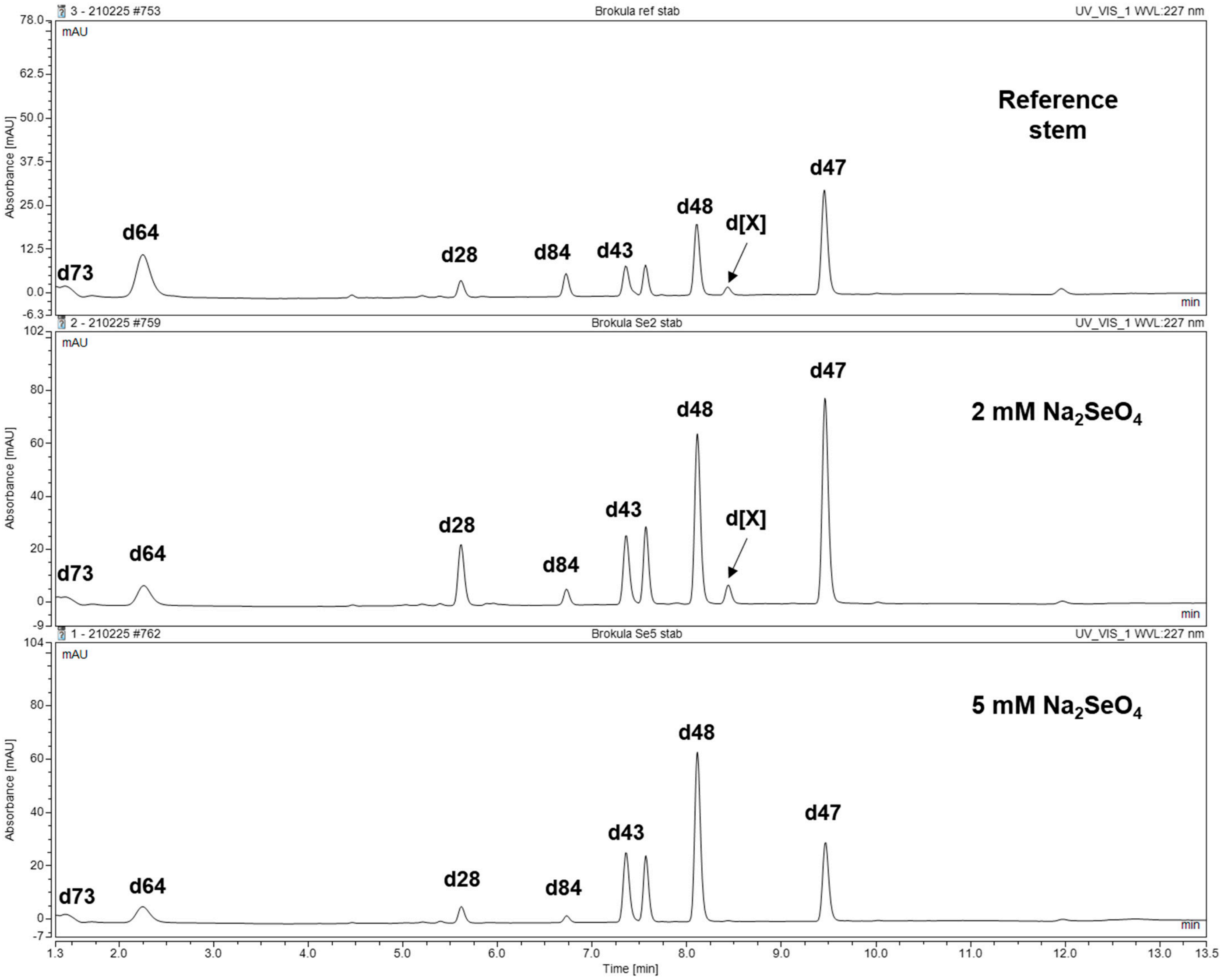

Figure S4. Chromatograms of desulfoglucosinolates (column temperature 25 °C) obtained from the stem of *Brassicca oleracea* var. *italica* before and after the treatment with Na_2SeO_4: **d73** – desulfoglucoiberin; **d64** – desulfoglucoraphanin; **d28** – desulfo-4-hydroxyglucobrassicin; **d84** – desulfoglucoerucin; **d43** – desulfoglucobrassicin; **d48** – desulfo-4-methoxyglucobrassicin; **d[X]** – hydroxymethoxydesulfoglucobrassicin; **d47** – desulfogluconeobrassicin.

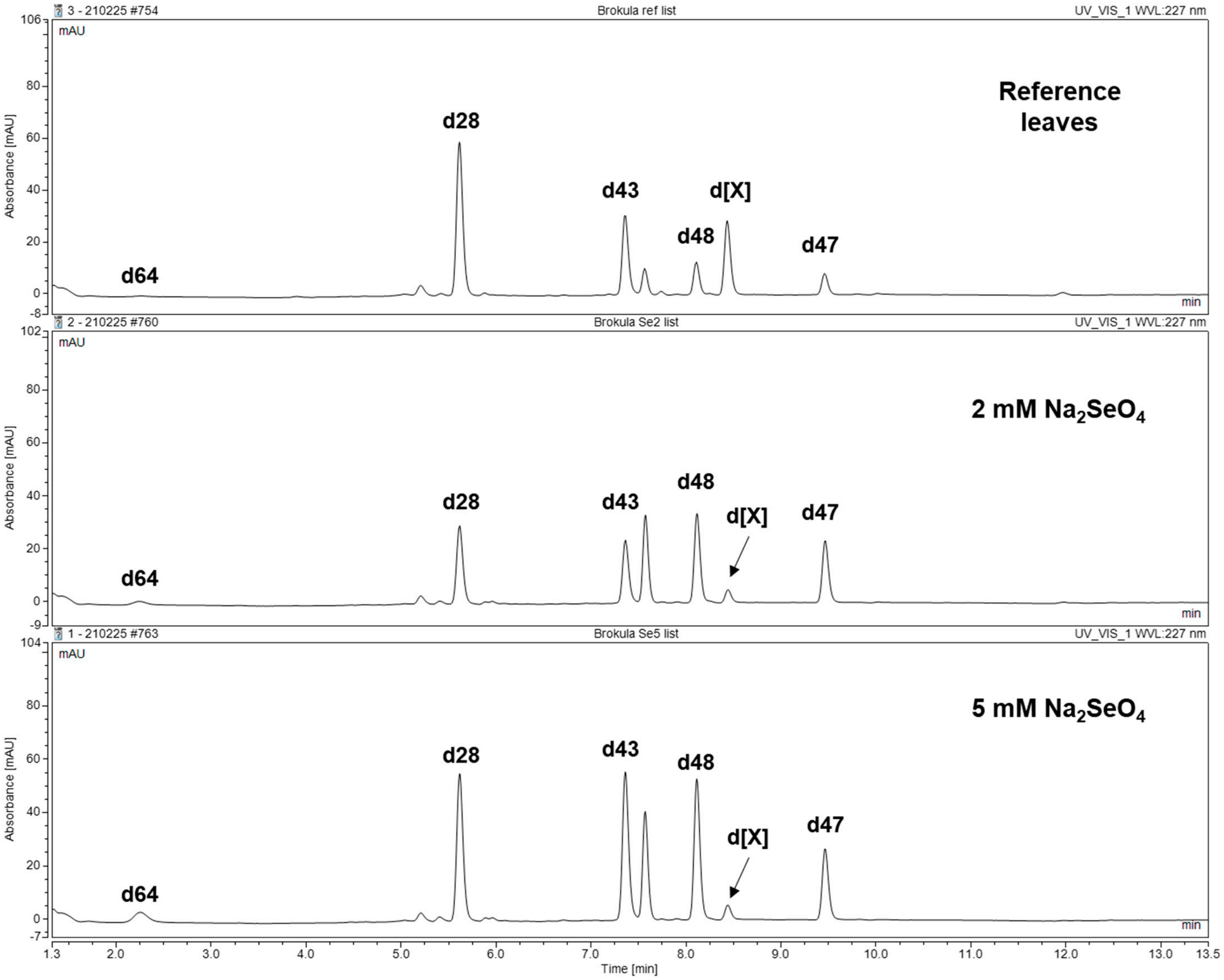

Figure S5. Chromatograms of desulfoglucosinolates (column temperature 25 °C) obtained from the leaf of *Brassicca oleracea* var. *italica* before and after the treatment with Na_2SeO_4: **d64** – desulfoglucoraphanin; **d28** – desulfo-4-hydroxyglucobrassicin; **d43** – desulfoglucobrassicin; **d48** – desulfo-4-methoxyglucobrassicin; **d[X]** – hydroxymethoxydesulfoglucobrassicin; **d47** – desulfogluconeobrassicin.

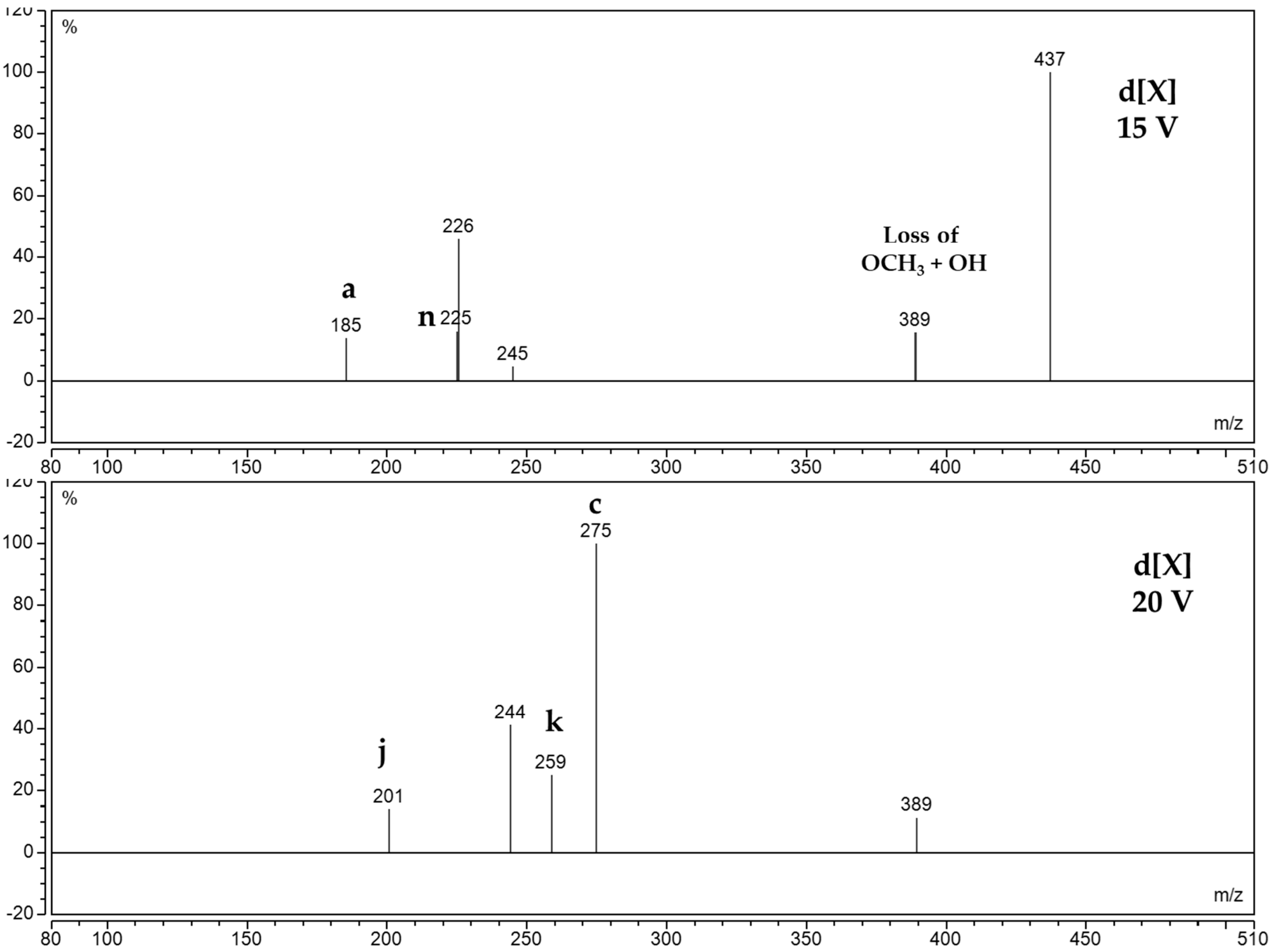

Figure S6. MS² spectra at 15V and 20V of sodium adduct of desulfo-hydroxymethoxyglucobrassicin [**dX**]. Fragment types observed, alone or in combination, in MS² spectra desulfoglucosinolates (dGSLs) in positive mode: a - Na^+ adduct of anhydroglucose, $C_6H_{10}O_5$ (at m/z 185) or an acyl derivative; c - Loss of anhydroglucose (m/z 162) or an acyl derivative; j - Na^+ adduct of gluconolactone; k - loss of gluconolactone (m/z 178). [1]

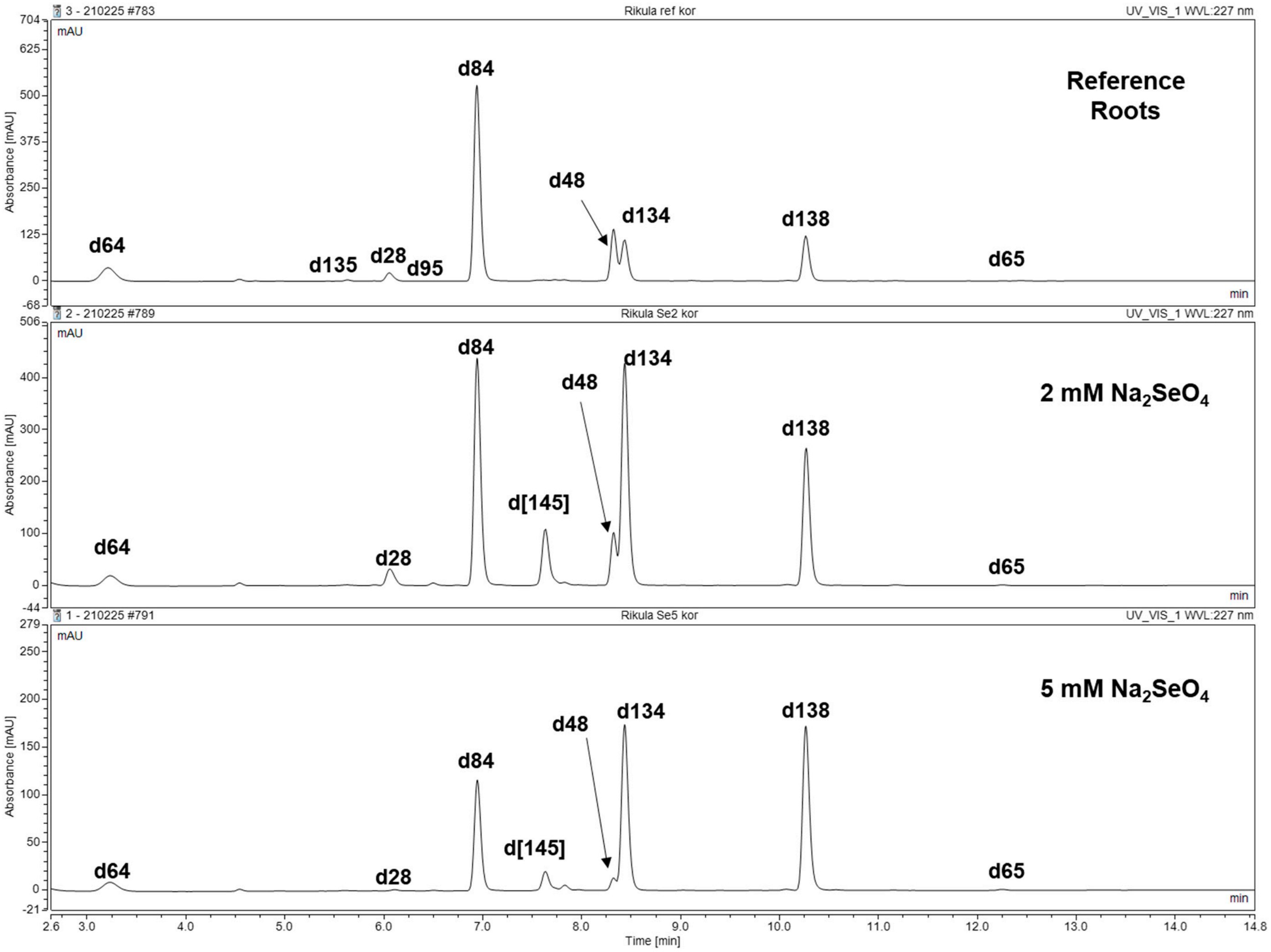

Figure S7. Chromatograms of desulfoglucosinolates (column temperature 15 °C) obtained from the roots of *Eruca vesicaria* before and after the treatment with Na_2SeO_4: **d64** – desulfoglucoraphanin; **d135** – 4-(*β*-D-glucopyranosyldisulfanyl)butyl dGSL; **d28** – desulfo-4-hydroxyglucobrassicin; **d95** – desulfoglucoibervirin; **d84** – desulfoglucoerucin; **d[145]** – desulfoglucoselenoerucin; **d48** – desulfo-4-methoxyglucobrassicin; **d134** – dimeric desulfo-4-mercaptobutyl GSL; **d138** – 1,4-desulfo-dimethoxyglucobrassicin; **d65** – desulfoglucocamelinin.

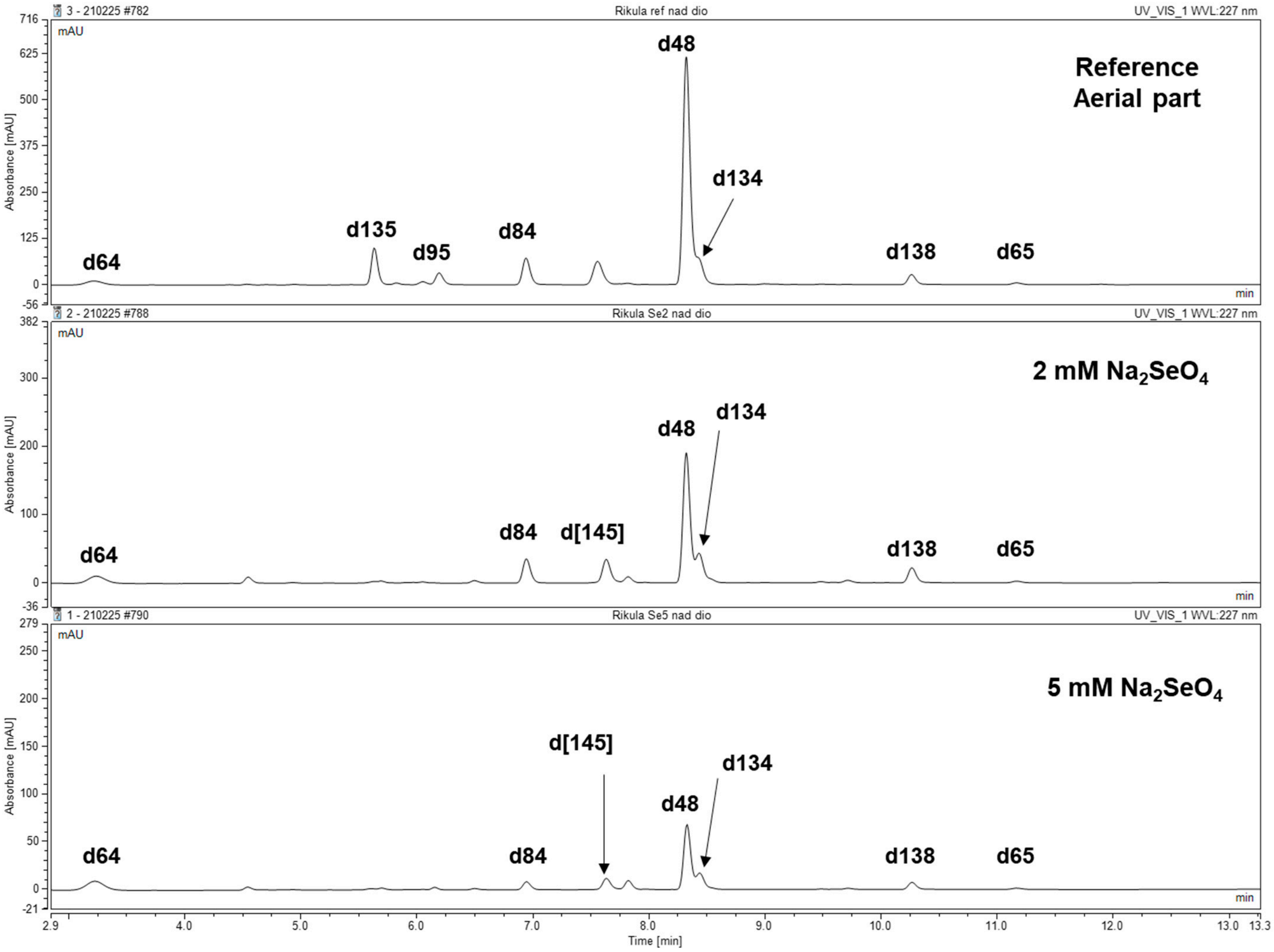

Figure S8. Chromatograms of desulfoglucosinolates (column temperature 15 °C) obtained from the aerial part of *Eruca vesicaria* before and after treatment with Na_2SeO_4: **d64** – desulfoglucoraphanin; **d135** – 4-(*β*-D-glucopyranosyldisulfanyl)butyl dGSL; **d95** – desulfoglucoibervirin; **d28** – desulfo-4-hydroxyglucobrassicin; **d95** – desulfoglucoibervirin; **d84** – desulfoglucoerucin; **d[145]** – desulfoglucoselenoerucin; **d48** – desulfo-4-methoxyglucobrassicin; **d134** – dimer of 4-mercaptobutyl dGSL; **d138** – 1,4-desulfo-dimethoxyglucobrassicin; **d65** – desulfoglucocamelinin.

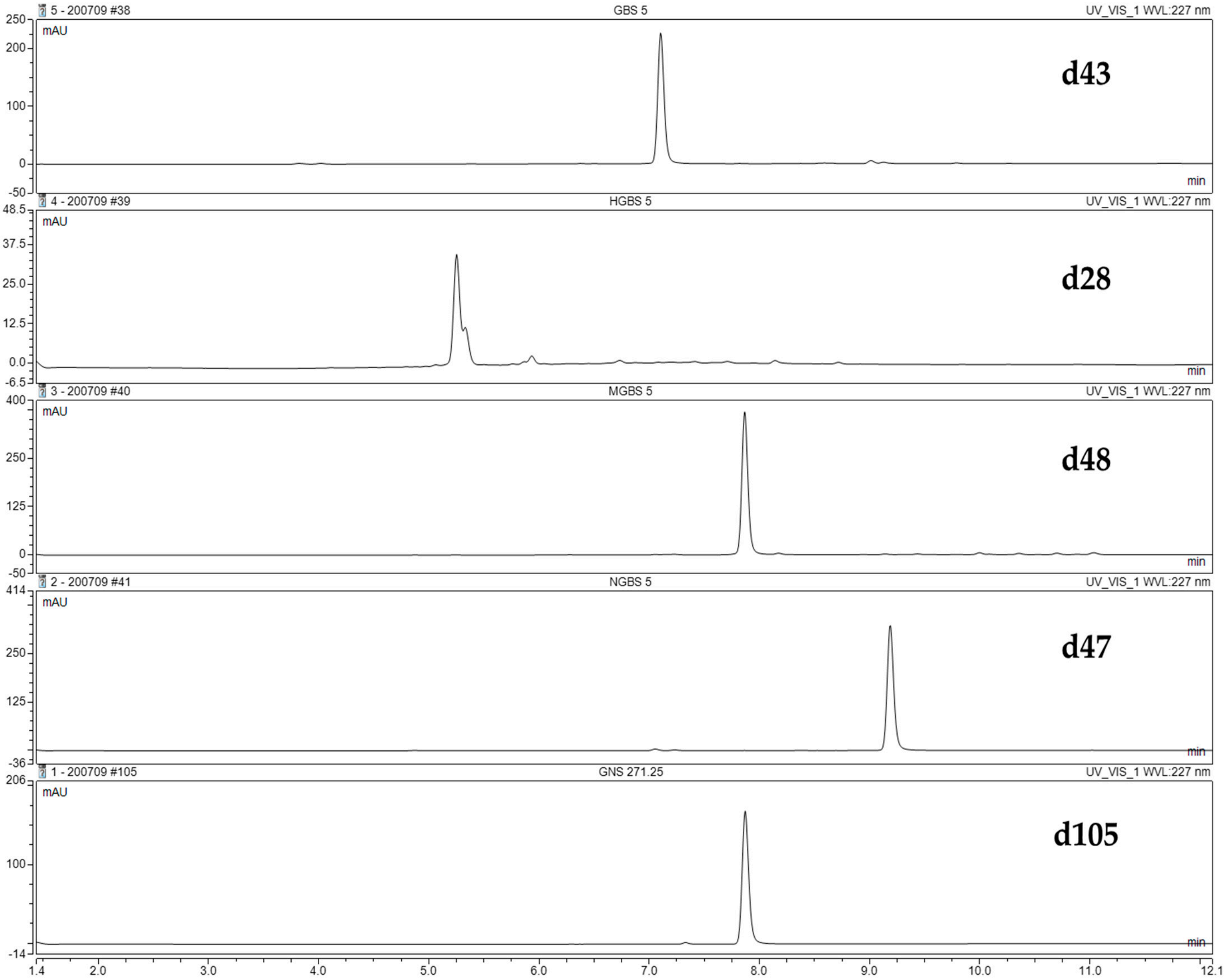

Figure S9. *Continuous*

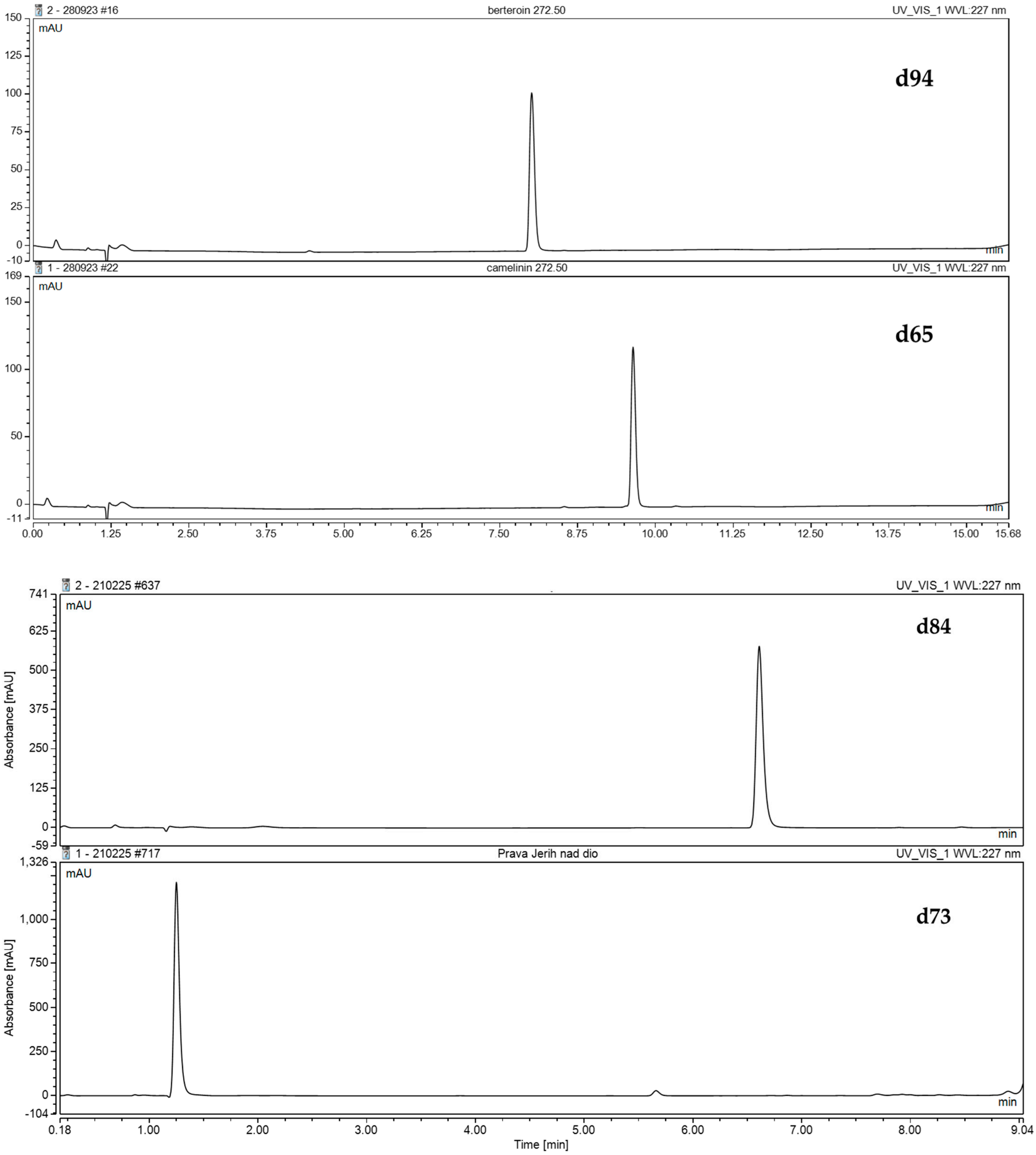

Figure S9. Chromatograms of desulfoglucosinolate (dGSL) standards. **d28** - 4-hydroxyindol-3-ylmethyl dGSL (desulfo-4-hydroxyglucobrassicin); **d43** - indol-3-ylmethyl dGSL (desulfoglucobrassicin); **d47** – *N*-methoxyindol-3-ylmethyl dGSL (desulfoneoglucobrassicin); **d48** – 4-methoxyindol-3-ylmethyl dGSL (desulfo-4-methoxyglucobrassicin); **d65** - 10-(methylsulfinyl)decyl dGSL (desulfoglucocamelinin); **d73** – 3-(methylsulfinyl)propyl dGSL (desulfoglucoiberin); **d84** – 4-(methylsulfanyl)butyl dGSL (desulfoglucoerucin); **d94** - 3-(methylsulfanyl)propyl dGSL(desulfoglucoibervirin); **d105** – phenylethyl dGSL (desulfogluconasturtiin).

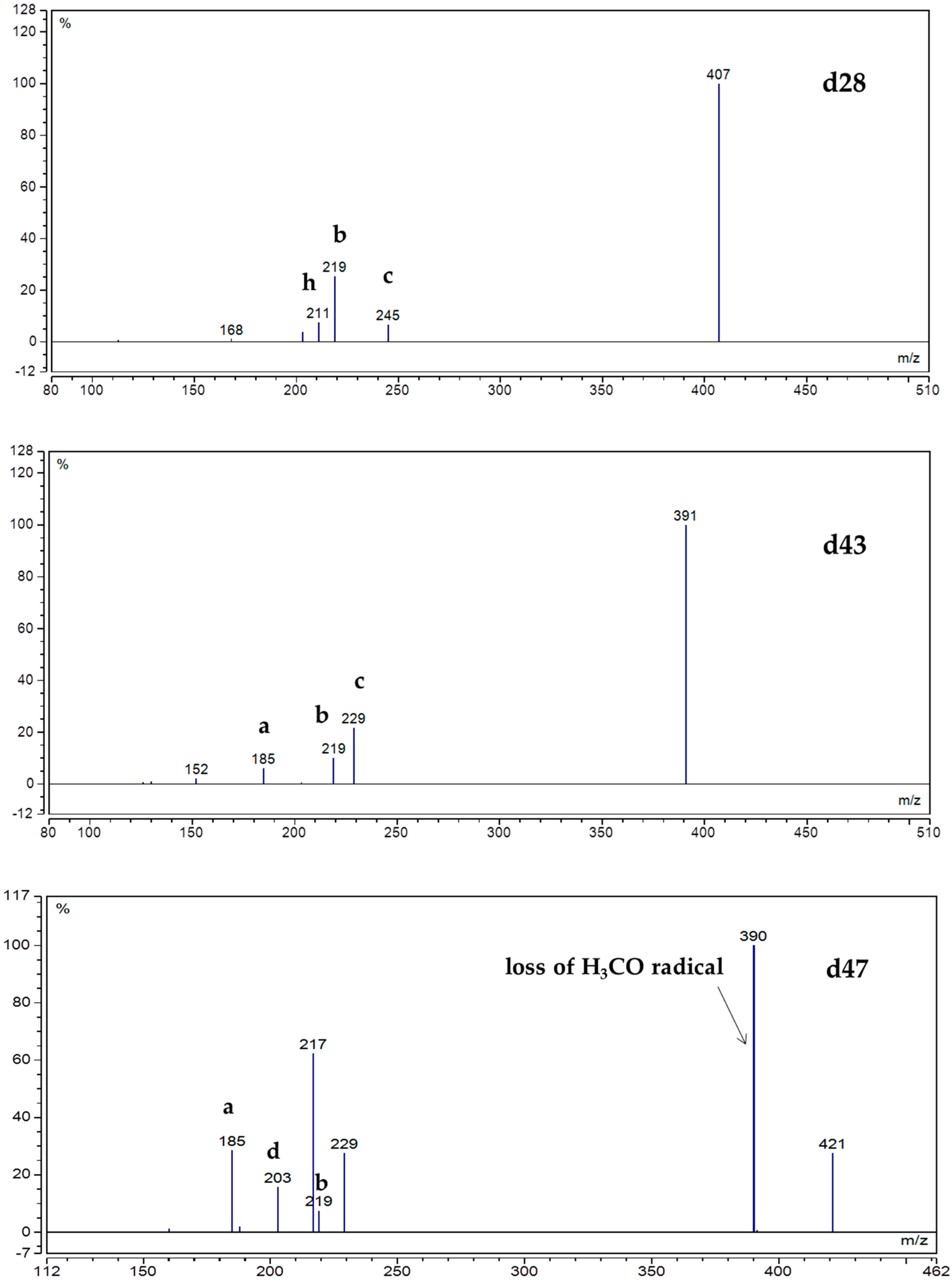

Figure S10. *Continuous*

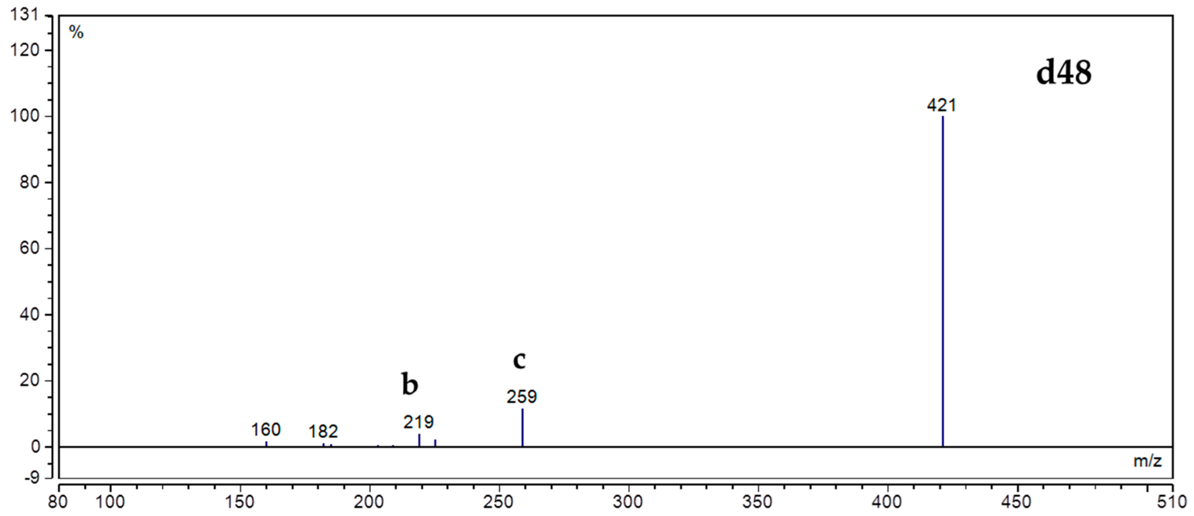

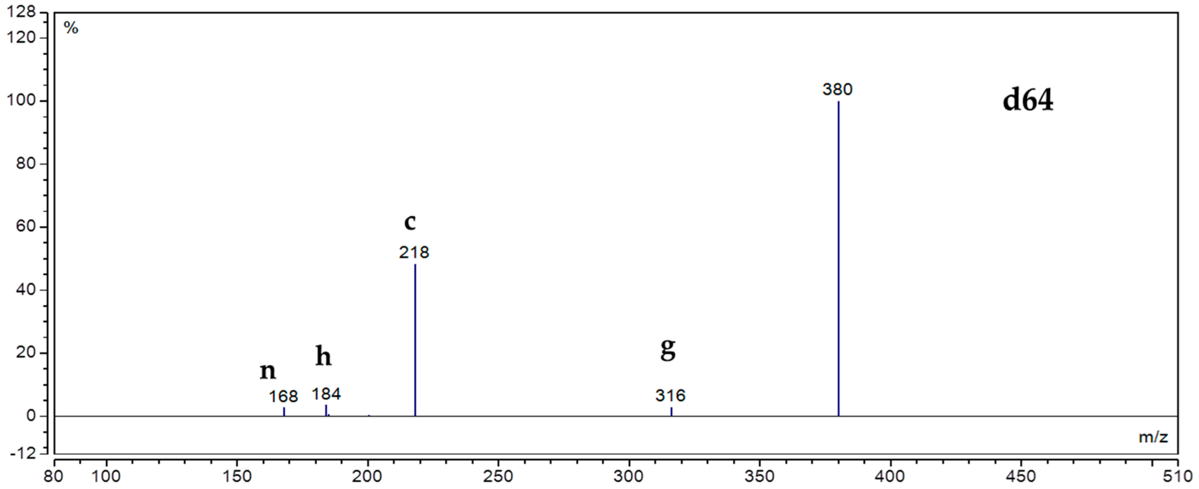

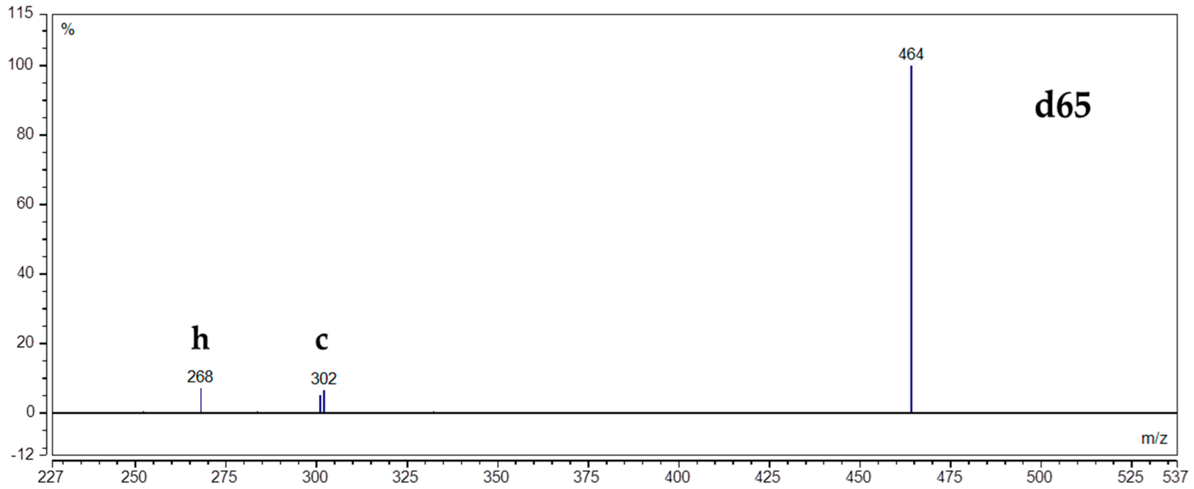

Figure S10. *Continuous*

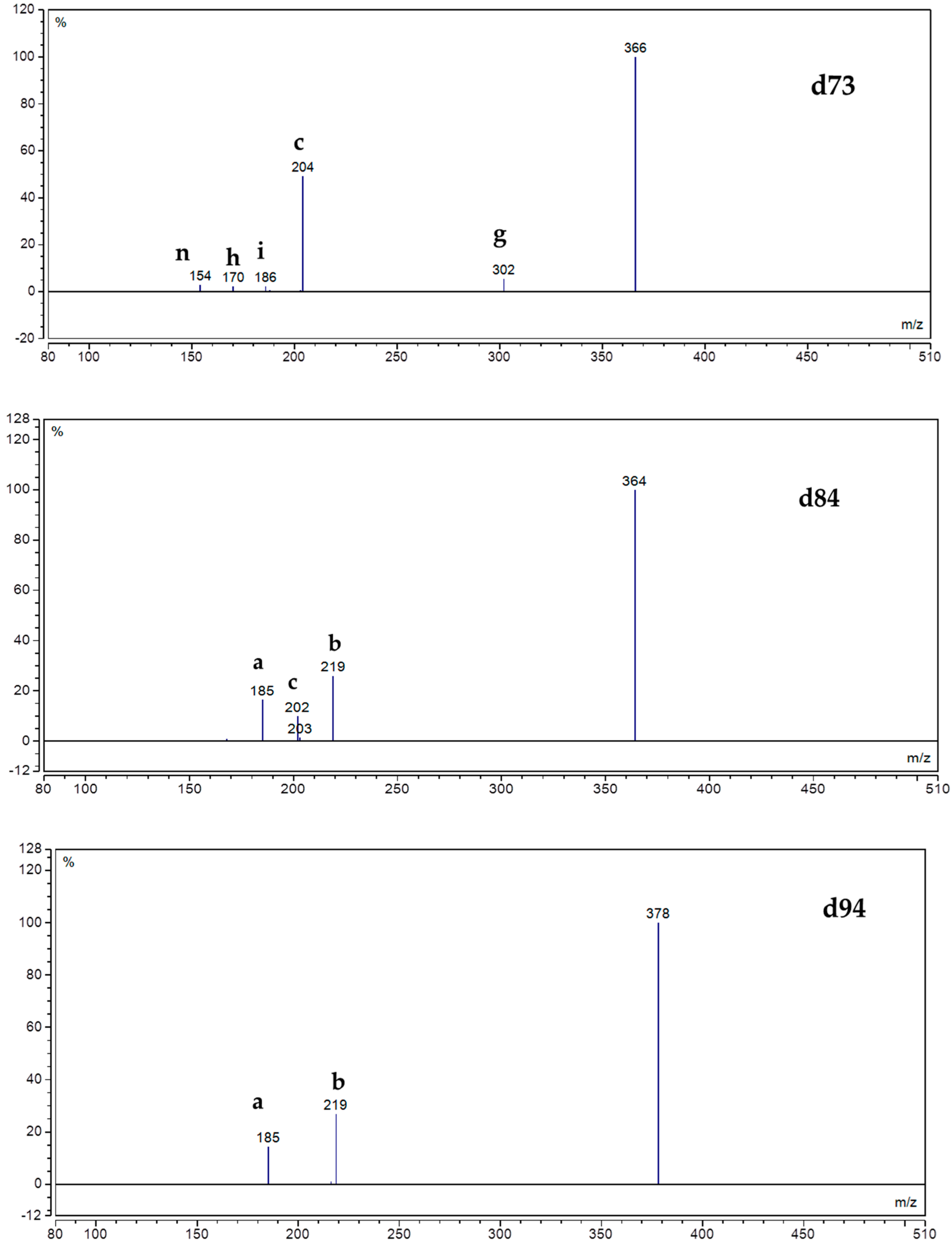

Figure S10. *Continuous*

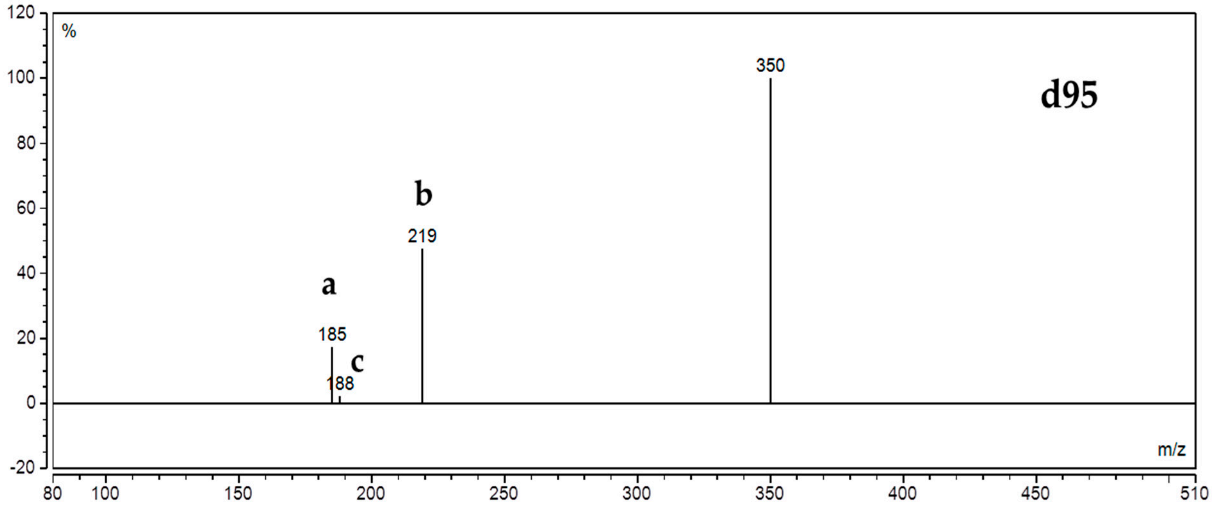

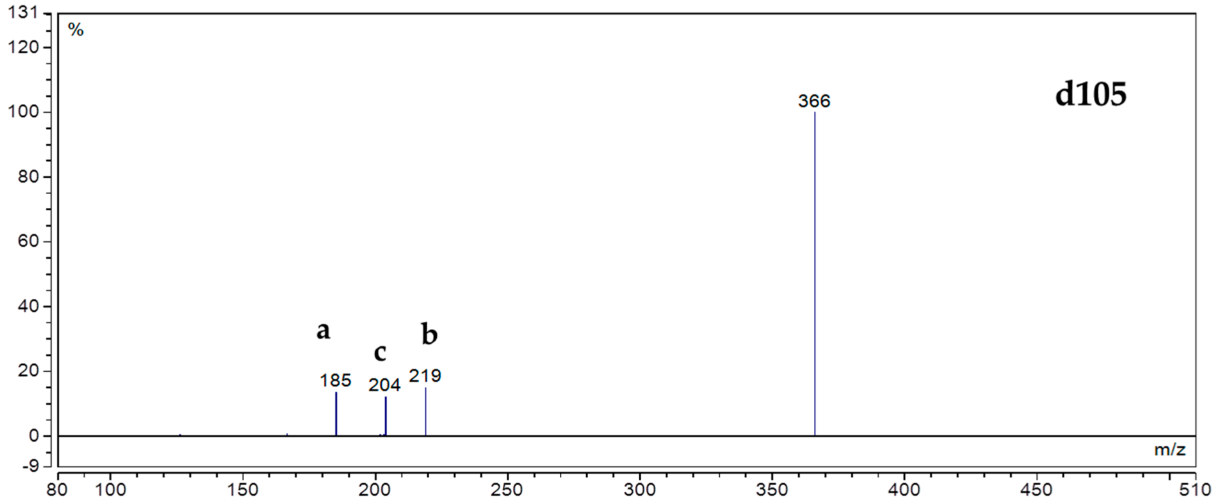

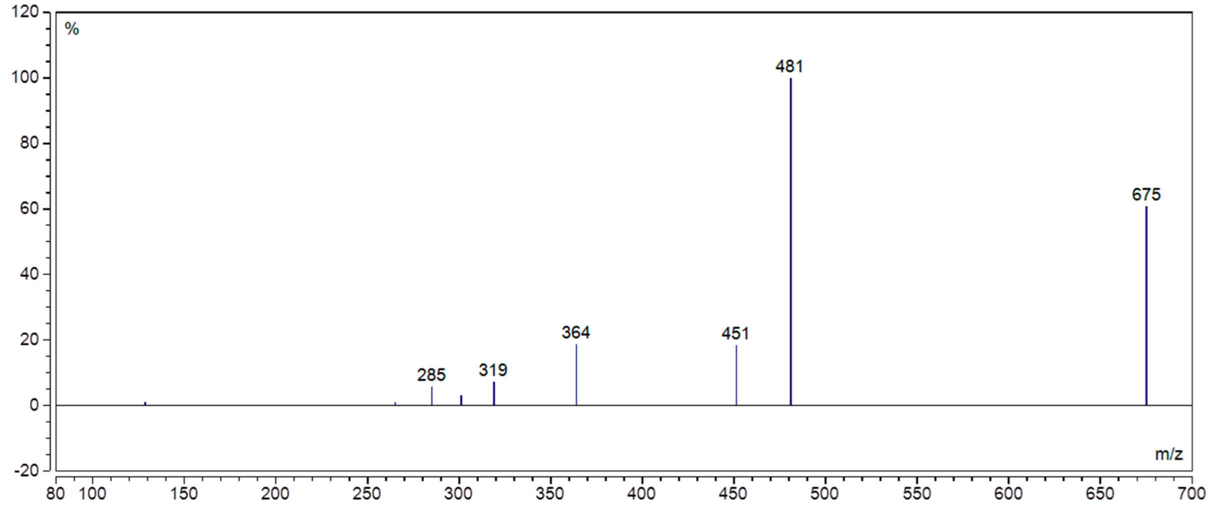

Figure S10. *Continuous*

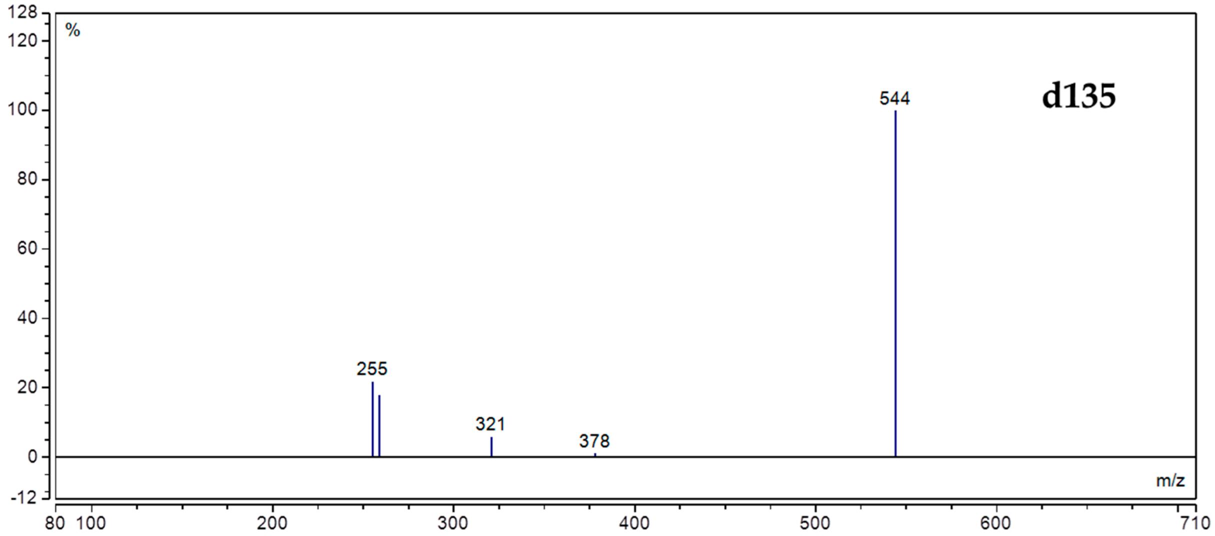

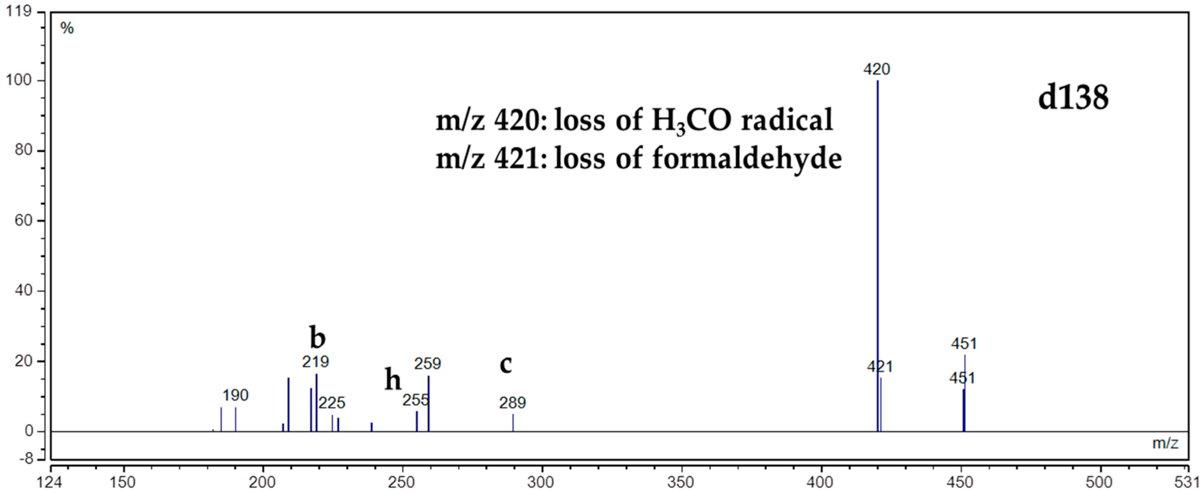

Figure S10. MS[2] spectra of sodium adduct of desulfoglucosinolates (dGSLs). **d28** - 4-hydroxyindol-3-ylmethyl dGSL (desulfo-4-hydroxyglucobrassicin); **d43** - indol-3-ylmethyl dGSL (desulfoglucobrassicin); **d47** – *N*-methoxyindol-3-ylmethyl dGSL (desulfoneoglucobrassicin); **d48** – 4-methoxyindol-3-ylmethyl dGSL (desulfo-4-methoxyglucobrassicin); **d64** – 4-(methylsulfinyl)butyl dGSL (desulfoglucoraphanin); **d65** - 10-(methylsulfinyl)decyl dGSL (desulfoglucocamelinin); **d73** – 3-(methylsulfinyl)propyl dGSL (desulfoglucoiberin); **d84** – 4-(methylsulfanyl)butyl dGSL (desulfoglucoerucin); **d94** – 3-(methylsulfanyl)propyl dGSL(desulfoglucoibervirin); 3-(methylsulfanyl)propyl dGSL (desulfoglucoiberivirin); **d105** – phenylethyl dGSL (desulfogluconasturtiin); **d134** – dimeric 4-mercaptobutyl dGSL; **d135** – 4-(*β*-D-glucopyranosyldisulfanyl)butyl dGSL (desulfodiglucothiobeinin); **d138** – 1,4-dimethoxyindol-3-ylmethyl dGSL (desulfo-1,4-dimethoxyglucobrassicin). Fragment types observed, alone or in combination, in MS[2] spectra desulfoglucosinolates in positive mode: **a** - Na^+ adduct of anhydroglucose, $C_6H_{10}O_5$ (at m/z 185) or an acyl derivative; **b** - Na^+ adduct of thioglucose, $C_6H_{11}O_5SH$ (at m/z 219) or an acyl derivative; **c** - Loss of anhydroglucose (m/z 162) or an acyl derivative; **d** - Na^+ adduct of glucose, $C_6H_{12}O_6$ (at m/z 203); **g** - loss of $H(SO)CH_3$ (m/z 64); **h** - loss of thioglucose (m/z 196); **i** - loss of glucose (m/z 180); **n** - loss of oxo-thioGlc (m/z 212). [1]

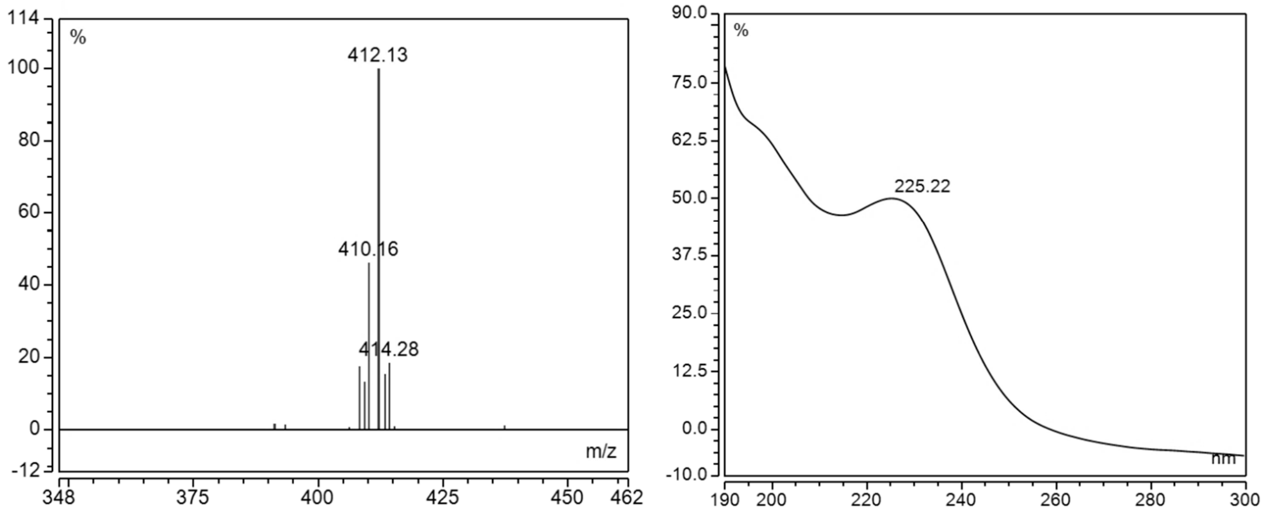

Figure S11. MS and UV spectra of detected sodium adduct of desulfoglucoselenoerucin **d[145]**. Two main $[M+Na]^+$ of isotopologues containing ^{78}Se and ^{80}Se can be observed at m/z 410 and 412.

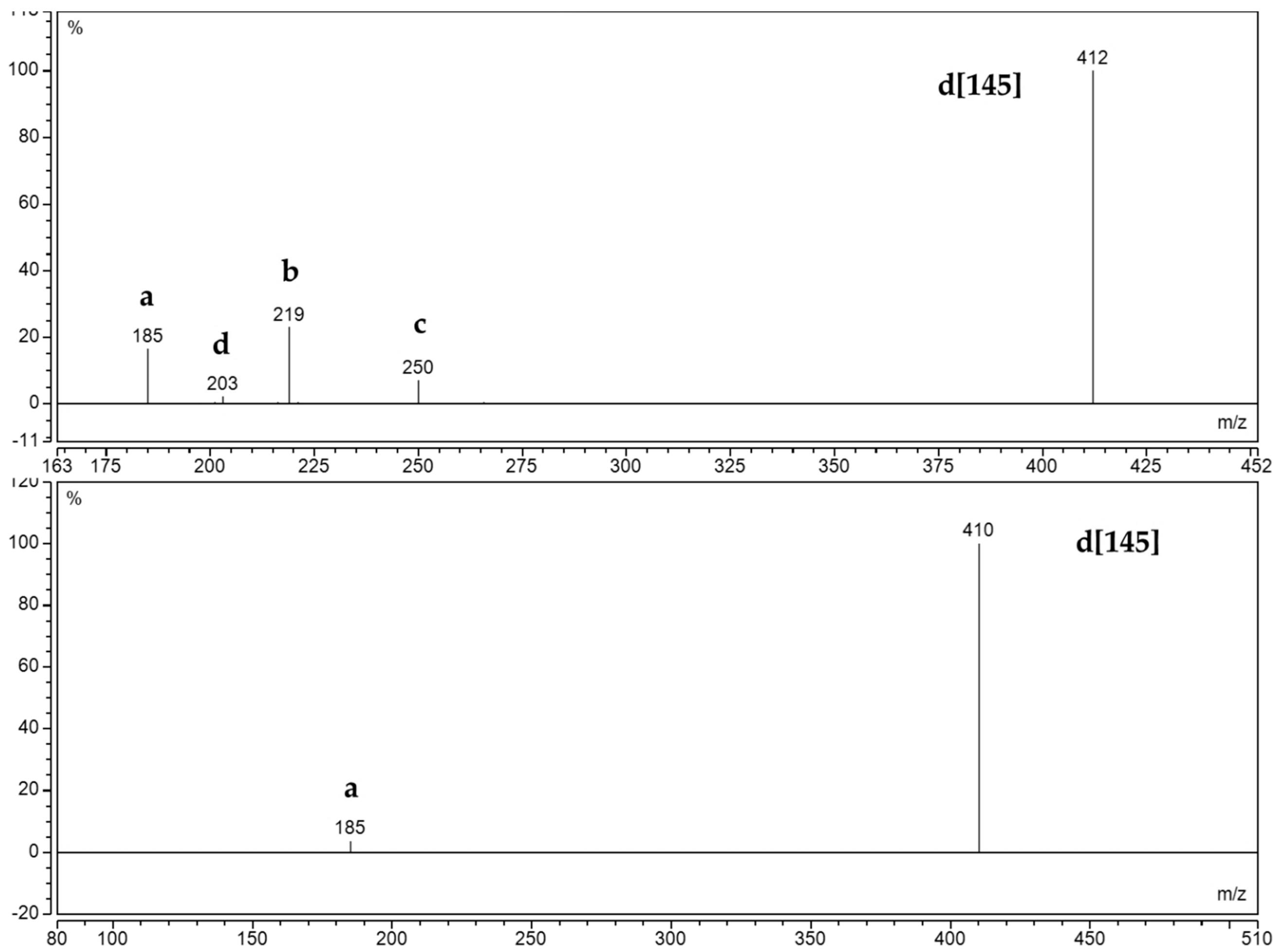

Figure S12. MS² spectra at 15V of sodium adduct of desulfoglucoselenoerucin (**d[145]**) formed during biofortification (two isotopes od Se - m/z 410 and 412).

References

1. Agerbirk, N.; Hansen, C.C.; Olsen, C.E.; Kiefer, C.; Hauser, T. P.; Christensen, S. et al. Glucosinolate profiles and phylogeny in *Barbarea* compared to other tribe Cardamineae (Brassicaceae) and *Reseda* (Resedaceae), based on a library of ion trap HPLC-MS/MS data of reference desulfoglucosinolates. *Phytochemistry*, **2021**, *185*, 112658 https://doi.org/10.1016/j.phytochem.2021.112658
